# Supplementary material for: Persistence and turnover of soil organic carbon in global drylands
Source: Nat Commun. 2026 Apr 22;17:3565. doi: 10.1038/s41467-026-70623-9 (PMC13103084; doi:10.1038/s41467-026-70623-9)
Supplement: Supplementary file 1 — Supplementary Information [file 41467_2026_70623_MOESM1_ESM.pdf]

Supplementary information for

# Persistence and turnover of soil organic carbon in global drylands

**The PDF file includes:**

Supplementary Text
Extended Data Figs.1 to 13
Extended Data Tables 1 to 14

## Supplementary Text

### The uncertainties of estimating the mean age of soil respired $CO_2$

While it is common to use a one-pool model to estimate the mean age of bulk SOC, as it predominantly reflects the old, slow-cycling C pool[1-3], it remains uncertain how to calculate the mean age (i.e., transit time) of respired C, since it is likely derived from multiple C pools with different cycling rates. This is also supported by our results that the $\Delta^{14}C$ values of respired $CO_2$ were consistently higher than those of bulk SOC. We estimated the mean age of the respired $CO_2$ based on a mass balance approach, assuming that the age of bulk SOC is estimated based on a one-pool model:

$$\text{age}_{\text{respired C}} = \text{age}_{\text{old C pool}} \times f_{\text{old}} + \text{age}_{\text{young C pool}} \times (1 - f_{\text{old}})$$

where $\text{age}_{\text{old C pool}}$ represents the mean age of bulk SOC calculated by the one-pool model, and $f_{\text{old}}$ is the proportion of old C in soil respired $CO_2$. The age of the bomb-derived young C was assumed to be 0. Using this approach, the mean age of respired C was 480 years (Extended Data Table 6).

### Two-pool models for estimating the mean transit time and system age

As described in the Materials and Methods, the two-pool model consists of a fast pool (Pool 1) and a slow pool (Pool 2), each with first order decomposition rates for SOM [4]. In the two-pool parallel model, C inputs are partitioned between the fast and slow pools, with a fraction $\gamma$ entering Pool 1 and $(1 - \gamma)$ entering Pool 2 (see the inset in the figure below). While in the two-pool series model, all C inputs first enter the fast pool, and a fraction of C is transferred from the fast to the slow-cycling pool (see the inset in the figure below). The model is assumed to be at steady state. The variables $C_1$ and $C_2$ represent the C stocks in Pool 1 and Pool 2, respectively. The model includes three adjustable parameters: $k_1$ ($yr^{-1}$), the decomposition rate of Pool 1, $k_2$ ($yr^{-1}$), the decomposition rate of Pool 2; and $a_{21}$ (unitless), the fraction of total C lost from Pool 1 (i.e., $k_1$ × Pool 1) that is transferred to Pool 2. Initial radiocarbon signatures for the model start year were calculated under steady-state conditions, using the equation:

$$\text{Fraction Modern} = k / (k + \lambda)$$

where $k$ is either $k_1$ or $k_2$, and $\lambda$ is the decay constant for radiocarbon (1/8267 $yr^{-1}$). The model was run from 1900 to 2020 with a time step of 0.1 years. We estimated parameters $k_1$, $k_2$, and $a_{21}$ from the observed $\Delta^{14}C$ in bulk and respired $CO_2$ together as described previously[4,5]. We run the models and estimated parameters for all data together (Overall, including Hyper Arid, Arid, Semi-Arid, and Dry sub-humid sites), but to be more illustrative, we also modeled all averaged Arid and Semi-Arid sites. Comparisons of the results from one-pool models ($1P_{bulk}$ and $1P_{inc}$), two-pool parallel ($2P_{Parallel}$) and two-pool series ($2P_{Series}$) models are provided in Extended Data Table 7.

Note that mean transit times are similar for one-pool and two-pool models (ranging from centuries to thousands of years), while the turnover times (inverse of the decay rates) of the fast pool in two-pool models are < 30 years. This indicates the importance of contributions from the slow pool to respired C and the non-homogeneous respiration sources.

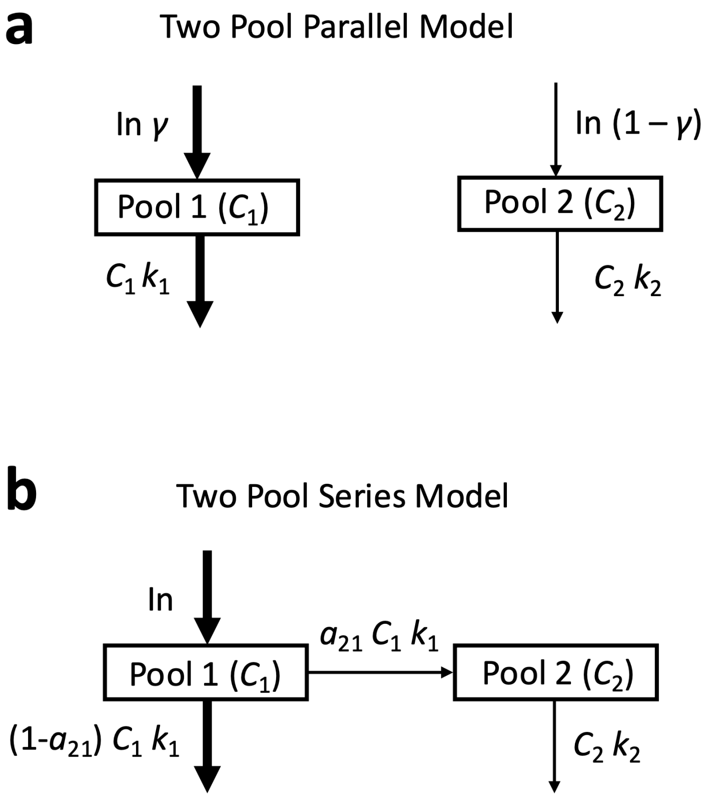


**Comparison of $\Delta^{14}C$ of respired $CO_2$ across regions**

In addition to our $\Delta^{14}C$ data, we extracted $\Delta^{14}C$ data of respired $CO_2$ from the International Soil Radiocarbon Database (ISRaD) based on the following selection criteria: (1) topsoil samples from 0–10 cm depth; (2) collected from natural or semi-natural ecosystems (cropland samples were excluded); (3) incubated under standardized control conditions (e.g., ~60% water holding capacity and temperatures representative of the growing season), excluding samples under treatments such as warming, increased precipitation, or nitrogen addition; (4) soils were root-picked to ensure that all respired $CO_2$ originated from microbes; and (5) incubation duration was less than 300 days to minimize alterations in microbial community structure and soil isotope signatures. In total, we obtained 352 observations of $\Delta^{14}C$ in respired $CO_2$ from ISRaD. To compare $\Delta^{14}C$ values between dryland and non-dryland ecosystems, we classified climate zones based on the aridity index (AI) as follows[6]: Hyper Arid (AI < 0.03), Arid (AI from 0.03 to 0.2), Semi-Arid (AI from 0.2 to 0.5), Dry sub-humid (AI from 0.5 to 0.65). Additionally, we included Sub-humid (AI from 0.65 to 1) and Humid (AI > 1) zones to compare. Results are presented in Extended Data Fig. 2.

### The potential influence of pH on soil $\Delta^{14}C$ characteristics and its uncertainties

We found contrasting effects of soil pH on the $\Delta^{14}C$ of bulk SOC and respired $CO_2$, although these trends were not statistically significant (Fig. 2; Extended Data Tables 9 and 10). Increasing soil pH was associated with lower $\Delta^{14}C$ values in bulk SOC, suggesting enhanced C persistence (Fig. 2a). This may be partly attributed to the accumulation of calcium carbonate ($CaCO_3$) and limited leaching under dry conditions[7,8]. Elevated $Ca^{2+}$ concentrations can promote the formation of cation bridges between organic molecules and mineral surfaces[9-11], thereby stabilizing SOC and reducing its turnover. In contrast, soil pH showed a positive correlation with the $\Delta^{14}C$ of respired $CO_2$ (Fig. 2b), indicating that more alkaline soils tend to respire younger C despite containing older bulk SOC.

Despite careful efforts to account for contributions from inorganic C, some uncertainty remains due to the presence of carbonates in a portion of our samples (Extended Data Table 8). These carbonates may have influenced the measured $\Delta^{14}C$ of respired $CO_2$, particularly in highly alkaline soils with high SIC content (e.g., soils on the Loess Plateau; thus, these sites were ultimately excluded from the analysis). Therefore, the observed relationships between pH and the $\Delta^{14}C$ of respired $CO_2$ should be interpreted with caution, and future research should further constrain the potential contributions of inorganic C to soil $CO_2$ efflux measurements.

### High clay and silt content promotes the protection of old C in dryland soils

Although higher clay and silt content is generally associated with improved water retention and increased pore space that support microbial activity[12,13], our results show a non-significant relationship between Clay + silt content and the $\Delta^{14}C$ of both bulk SOC and respired $CO_2$ (Fig. 2; Extended Data Tables 9 and 10). This is probably because soils with higher clay and silt content (i.e., finer-textured soils) have greater specific surface area and surface charge, which enhance the adsorption of organic matter onto mineral surfaces and protect older C from microbial decomposition[14]. As a result, soils with higher clay and silt content may receive more fresh C input but also protect old C from decomposition, thereby confounding the $\Delta^{14}C$ signatures of bulk SOC and respired $CO_2$.

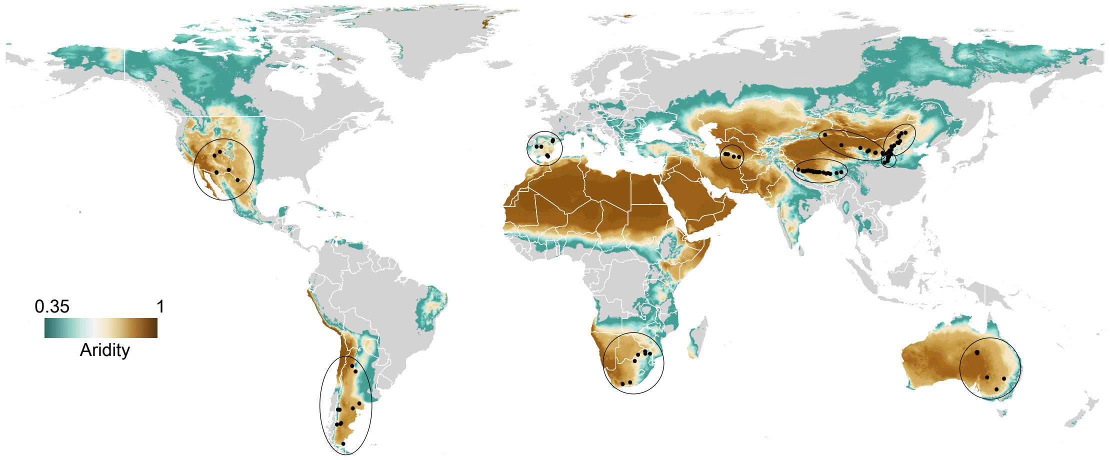


**Extended Data Fig. 1 | Locations of the dryland sites for paired Δ$^{14}$C of bulk SOC and respired $CO_2$.** Dryland areas are characterized by aridity levels (1 – aridity index) higher than 0.35, where aridity index is calculated as the ratio of mean annual precipitation to mean annual potential evapotranspiration[15,16]. The background of the global map represents the degree of aridity, with higher values indicating more arid regions. The hollow circles indicate samples collected from ten different regions worldwide. Further details about the surveyed field sites are provided in Extended Data Table 8.

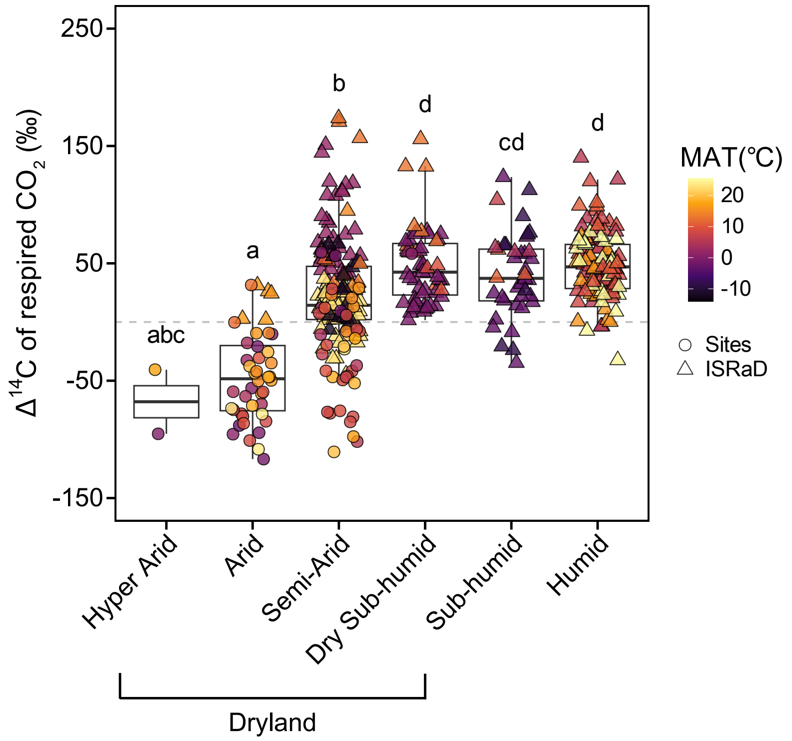


**Extended Data Fig. 2 | Characteristics of $\Delta^{14}C$ values of respired $CO_2$ across different aridity categories.** In this nomenclature, 0‰ corresponds to the preindustrial atmospheric baseline. Positive values ($\Delta^{14}C > 0‰$) indicate the presence of bomb-derived $^{14}C$ produced by atmospheric nuclear weapons testing and fixed within the past c. 60 years, whereas negative values ($\Delta^{14}C < 0‰$) indicate that C has remained in the soil long enough to undergo significant radioactive decay ($^{14}C$ half-life = 5730 years). Sampling included $n$ = 80 sites surveyed in this study, compared with $n$ = 352 entries from the International Soil Radiocarbon Database (ISRaD). Box plots show the median (center line) and the interquartile range (box, from the lower to the upper quartile), with whiskers extending to 1.5 times the interquartile range. Aridity index (AI) classes were defined as follows: Hyper-arid, AI < 0.05; Arid, 0.05 ≤ AI < 0.2; Semi-arid, 0.2 ≤ AI < 0.5; Dry sub-humid, 0.5 ≤ AI < 0.65; Sub-humid, 0.65 ≤ AI < 1; Humid, AI ≥ 1. Our sampling greatly expands the available $\Delta^{14}C$ data for arid and semi-arid regions and fills a previous gap for hyper-arid zones. Differences among climate groups were evaluated using Dunn's multiple comparison test.

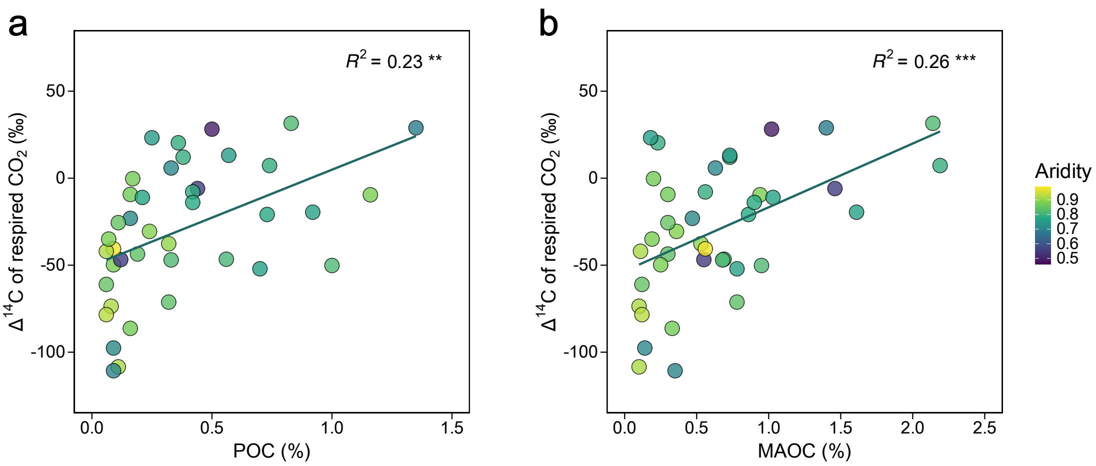


**Extended Data Fig. 3 | Relationship between Δ$^{14}$C of respired $CO_2$, $f_{old}$, and SOC fractions.** (**a**) Δ$^{14}$C of respired $CO_2$ vs. particulate organic carbon (POC) ($R^2$ = 0.23, $P$ < 0.01, $n$ = 41). (**b**) Δ$^{14}$C of respired $CO_2$ vs. mineral associated organic carbon (MAOC) ($R^2$ = 0.26, $P$ < 0.001, $n$ = 41). These patterns indicate that the older C contributing to respiration is not related to SOC fractionation (POC vs. MAOC). Significance levels: ** $P$ < 0.01, and *** $P$ < 0.001.

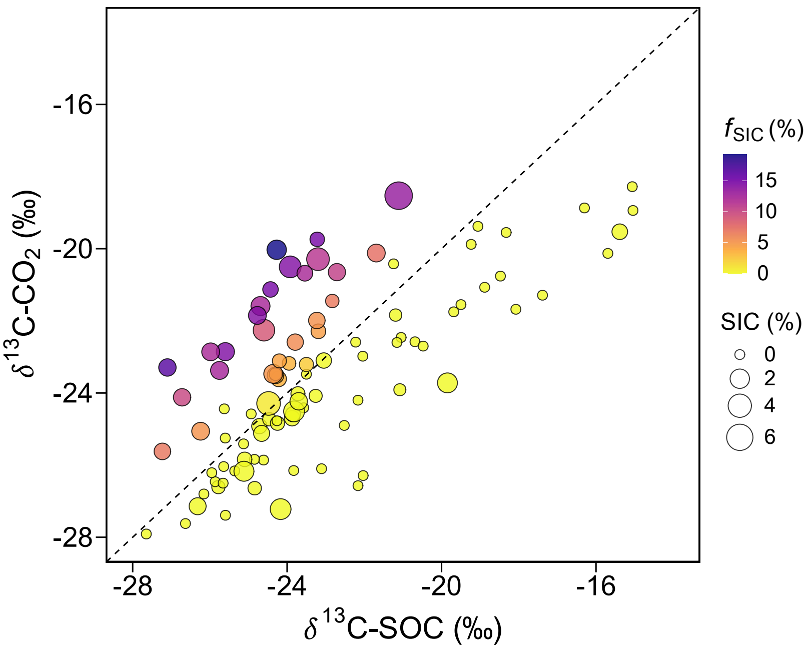


**Extended Data Fig. 4 | Relationship between the $\delta^{13}$C of respired $CO_2$ and bulk SOC.** $f_{SIC}$, the proportion of respired $CO_2$ derived from SIC, calculated based on a mass balance approach (see Material and methods). The dotted black line represents the 1:1 line. Data points with $f_{SIC} = 0$ are mostly located near or below the 1:1 line, suggesting that the $\delta^{13}$C of respired $CO_2$ is similar to or slightly lighter than that of bulk SOC. In contrast, as $f_{SIC}$ increases, points deviate above the 1:1 line, indicating that respired $CO_2$ becomes increasingly enriched in $\delta^{13}$C. This reflected increasing contributions from SIC to released $CO_2$ with increasing fractions of SIC.

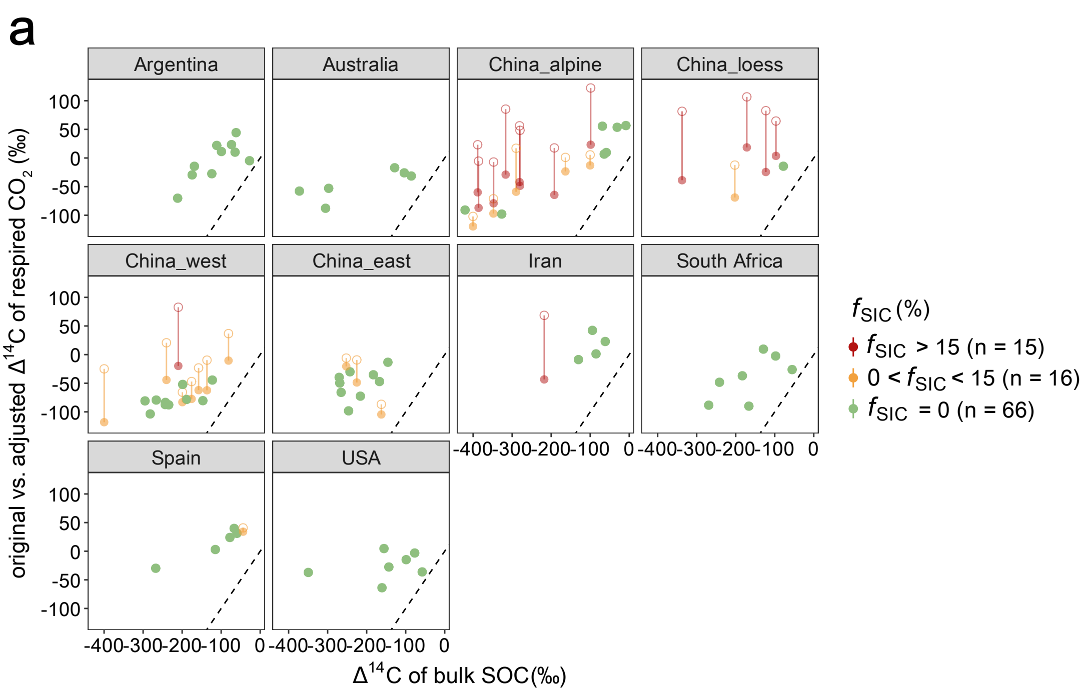


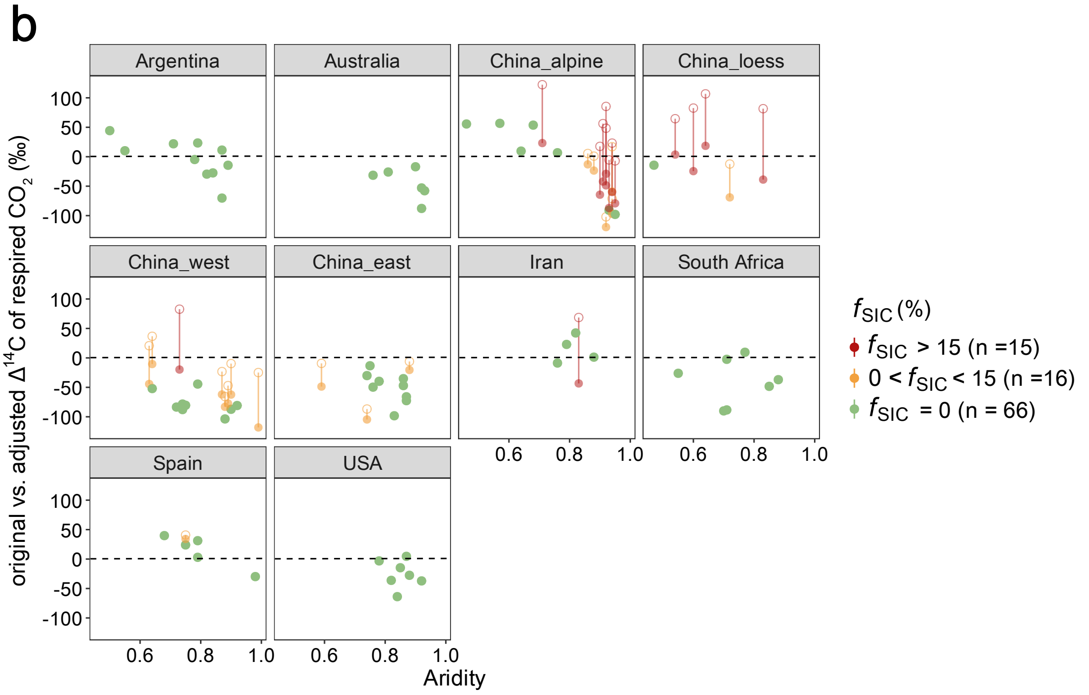


**Extended Data Fig. 5 | Characteristics of the original $\Delta^{14}C$ of respired $CO_2$ vs. the $\Delta^{14}C$ of respired $CO_2$ adjusted for the estimated fraction of SIC in released $CO_2$ ($f_{SIC}$).** (**a**) Relationships between original vs. adjusted $\Delta^{14}C$-$CO_2$, and bulk $\Delta^{14}C$. (**b**) Relationships between original vs. adjusted $\Delta^{14}C$-$CO_2$, and aridity. Filled cycles represent the original $\Delta^{14}C$-$CO_2$ from soil respired $CO_2$, and open cycles represent the $\Delta^{14}C$-$CO_2$ adjusted for $f_{SIC}$. We removed sites ($n$ = 15) with $f_{SIC}$ > 15% given on considering the uncertainty in $\Delta^{14}C$ of respired $CO_2$, and the 2 remaining sites located in the Loess Plateau. As a result, total of 80 sites were selected for the analysis of respired $\Delta^{14}C$.

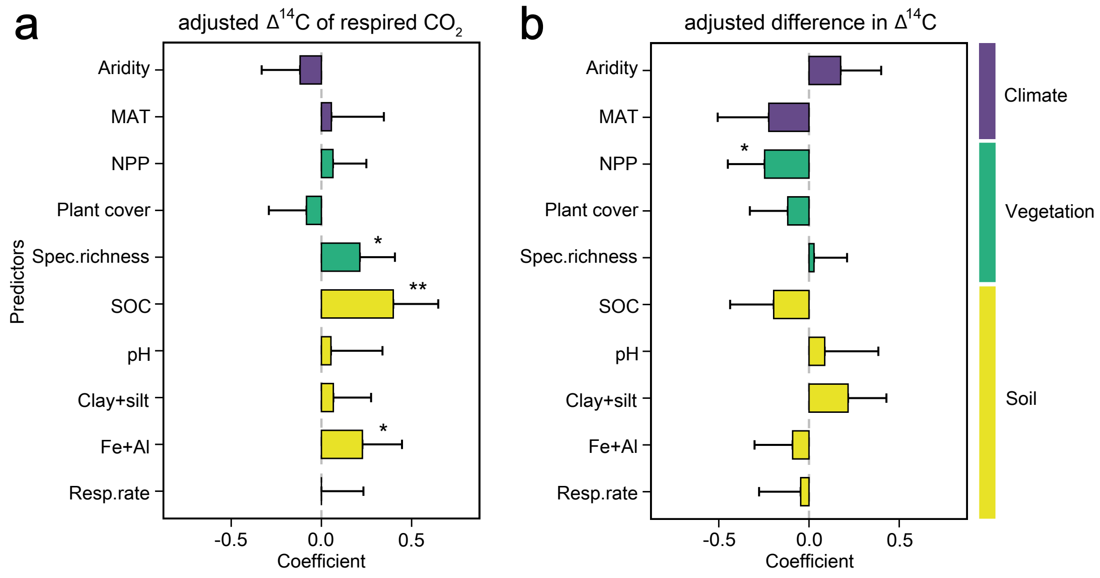


**Extended Data Fig. 6 | Predictors of the Δ$^{14}$C of respired $CO_2$ adjusted for the estimated fraction of SIC ($f_{SIC}$; sites where $f_{SIC}$ < 15%, $n$ = 80) based on linear mixed-effects regression models.** (**a**) Adjusted Δ$^{14}$C of respired $CO_2$ ($R^2$ = 0.57). (**b**) Adjusted difference in Δ$^{14}$C between bulk SOC and respired $CO_2$ ($R^2$ = 0.46). Linear mixed-effects regression models include climate variables (aridity [1 – aridity index] and mean annual temperature [MAT]), vegetation variables (net primary productivity [NPP], plant cover, and species richness [Spec. richness]), and soil properties (SOC, pH, clay + silt content [Clay + silt], oxalate-extractable Fe and Al oxides [Fe + Al], and microbial respiration rate [Resp. rate]). Error bars show 95% confidence intervals (CIs) of fixed-effect coefficients. Significance levels: * $P < 0.05$, and ** $P < 0.01$. $R^2$ values are conditional, representing variance explained by both fixed and random effects. SOC and NPP were consistently the most important explanatory variables for respired Δ$^{14}$C, and the differences between bulk and respired Δ$^{14}$C values, respectively, regardless of whether the Δ$^{14}$C values were adjusted by $f_{SIC}$ or not.

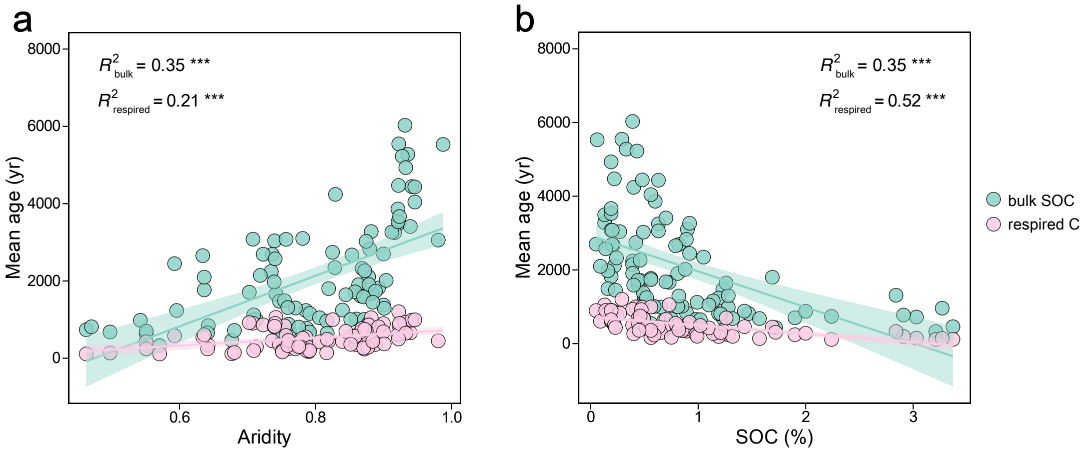


**Extended Data Fig. 7 | Mean age estimates of bulk SOC and respired C based on a one-pool model.** (**a**) Linear relationship between mean ages and aridity. (**b**) Linear relationship between mean ages and SOC content. The mean age of bulk SOC was 2100 ± 140 years (mean ± SE; *n* = 97), and the mean age (transit time) of respired C was 520 ± 30 years estimated by one-pool model (mean ± SE; *n* = 80). Both the mean age of bulk SOC and the transit time of respired C were positively correlated with aridity and negatively correlated with SOC content. Shaded areas represent the 95% confidence intervals of the fitted regression lines. Significance levels: *** $P < 0.001$.

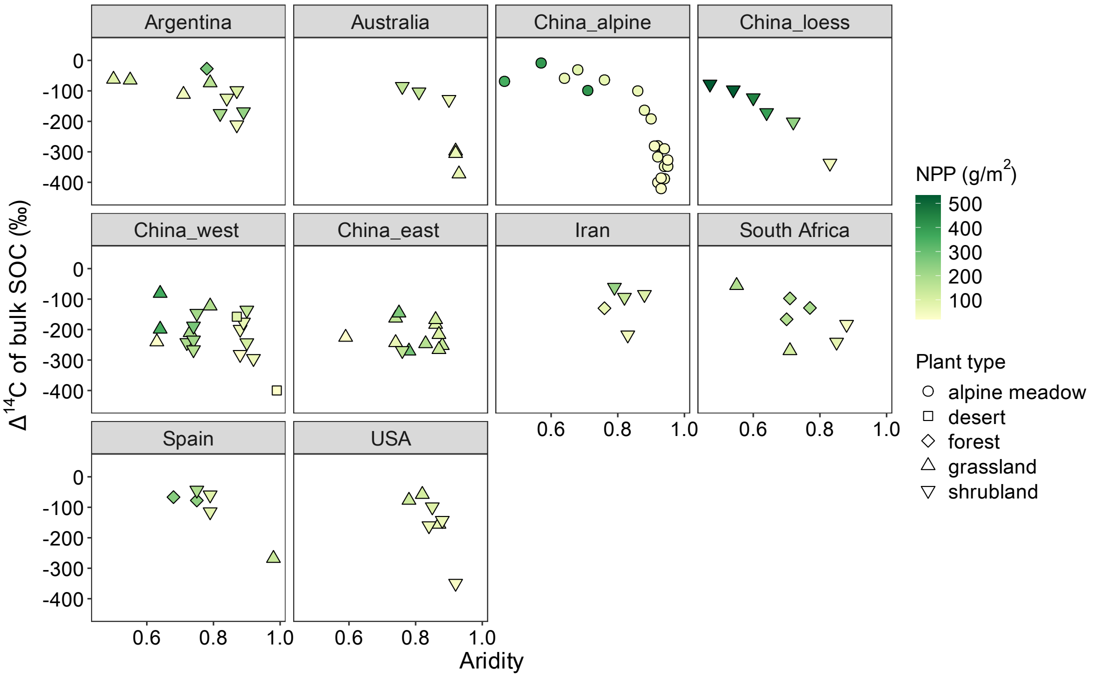


**Extended Data Fig. 8 | Variation in Δ$^{14}$C of bulk SOC from ten dryland regions along an aridity gradient.** Data points ($n$ = 97) are colored by net primary productivity (NPP) and shaped by plant type. In most regions, Δ$^{14}$C values of bulk SOC decreased with increasing aridity and decreasing NPP.

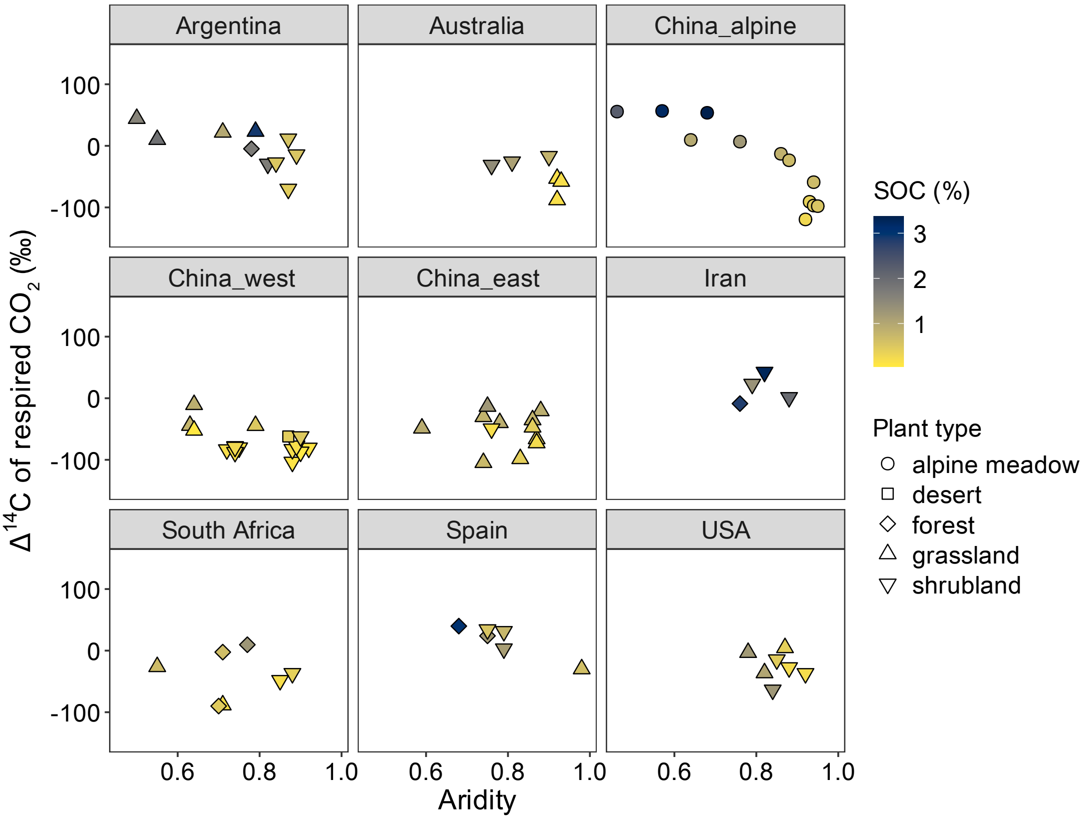


**Extended Data Fig. 9 | Variation in $\Delta^{14}C$ of soil respired $CO_2$ across ten dryland regions along an aridity gradient.** Data points ($n$ = 80) are colored by SOC content and shaped by plant type. In most regions, $\Delta^{14}C$ values of soil respired $CO_2$ decreased with increasing aridity.

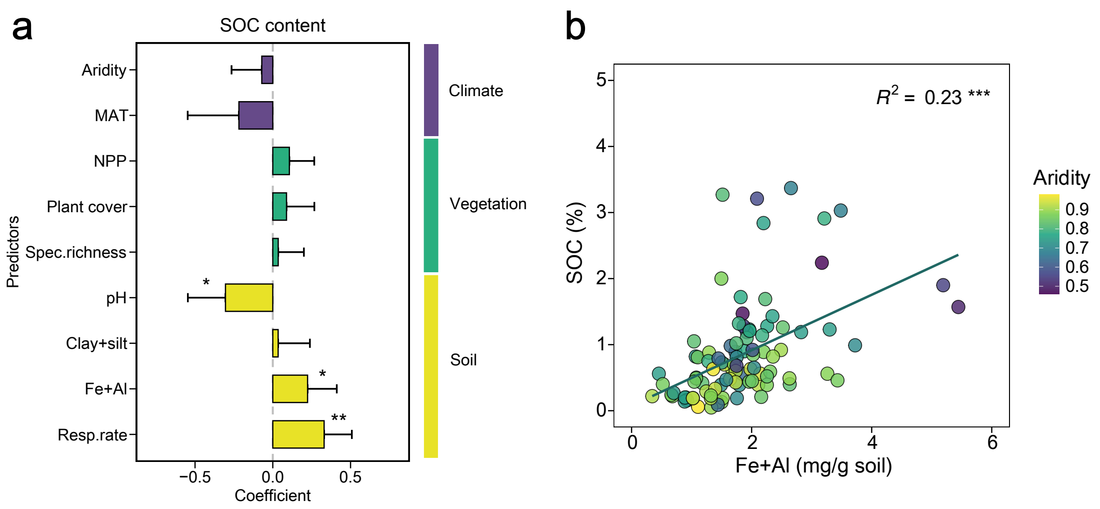


**Extended Data Fig. 10 | Factors affecting soil organic carbon (SOC) content across global drylands.** (**a**) Predictors of SOC content based on linear mixed-effects regression models. Linear mixed-effects regression models include climate variables (aridity [1 – AI] and mean annual temperature [MAT]), vegetation variables (net primary productivity [NPP], plant cover, and species richness [Spec. richness]), and soil properties (pH, clay + silt content [Clay + silt], oxalate-extractable Fe and Al oxides [Fe + Al], and microbial respiration rate [Resp. rate]). Conditional $R^2$ explained by the fixed and random effects is 0.60. (**b**) The linear relationship between SOC and oxalate-extractable Fe and Al. Error bars show 95% confidence intervals (CIs) of fixed-effect coefficients. Significance levels: * $P < 0.05$, ** $P < 0.01$, and *** $P < 0.001$. SOC content was negatively correlated with pH, but positively correlated with oxalate-extractable Fe and Al, as well as soil respiration rate. These results suggested that lower pH and greater availability of reactive Fe and Al may enhance SOC stabilization, while higher microbial activity implied a larger proportion of active or young C pools in the soil.

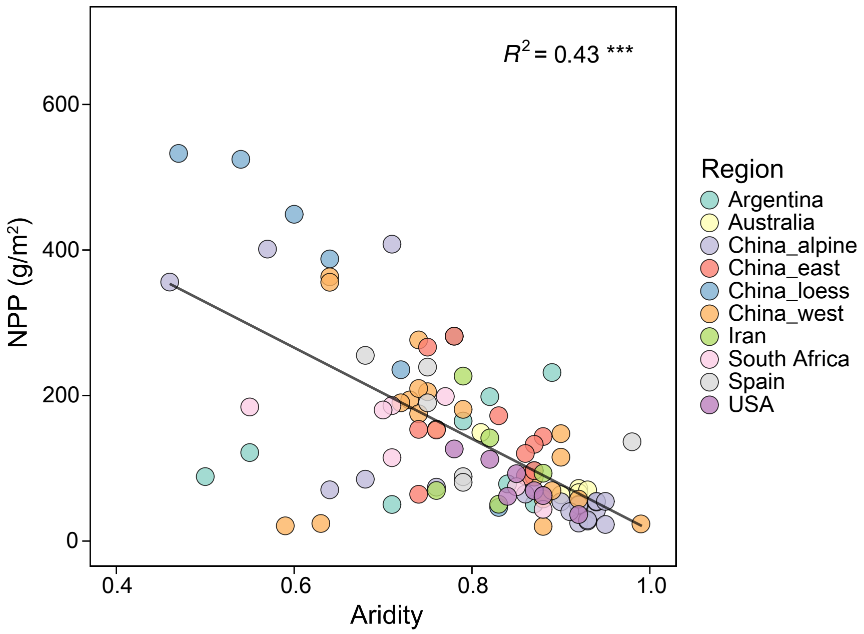


**Extended Data Fig. 11 | Relationship between NPP and aridity across regions.** The significant negative relationship between NPP and aridity ($R^2$ = 0.43) suggested water availability limits vegetation productivity in drylands. Significance levels: *** $P$ < 0.001.

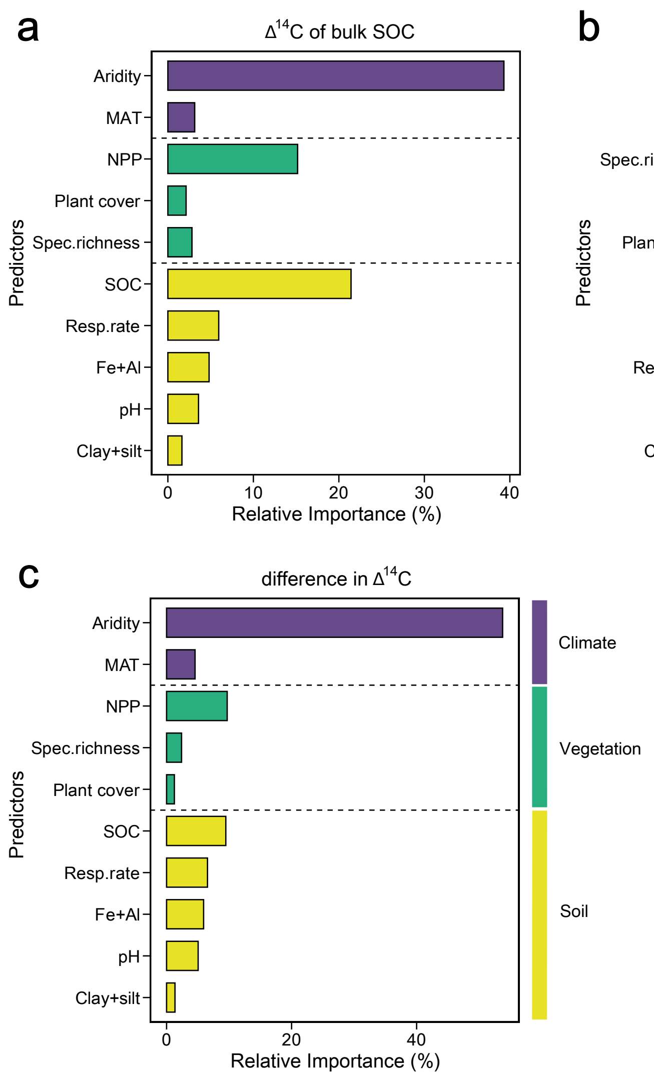


**Extended Data Fig. 12 | Predictors of radiocarbon signatures in global dryland soils based on random forest models.** (**a**) $\Delta^{14}C$ of bulk SOC ($n$ = 97, $R^2$ = 65.1%). (**b**) $\Delta^{14}C$ of respired $CO_2$ ($n$ = 80, $R^2$ = 41.3%). (**c**) $\Delta^{14}C$ difference between bulk SOC and respired $CO_2$ ($n$ = 80, $R^2$ = 35.0%). Random forest models include climate variables (aridity [1 – aridity index] and mean annual temperature [MAT]), vegetation variables (net primary productivity [NPP], plant cover, and species richness [Spec. richness]), and soil properties (SOC, pH, clay + silt content [Clay + silt], oxalate-extractable Fe and Al oxides [Fe + Al], and microbial respiration rate [Resp. rate]). $R^2$ refers to the model's ability to explain the variation in the response variable. Results from the random forest model were consistent with those from the linear mixed-effects regression models (Fig. 2). Specifically, aridity, NPP, and SOC were the most important explanatory variables for $\Delta^{14}C$ of bulk SOC, while SOC was the most important factor for $\Delta^{14}C$ of respired $CO_2$.

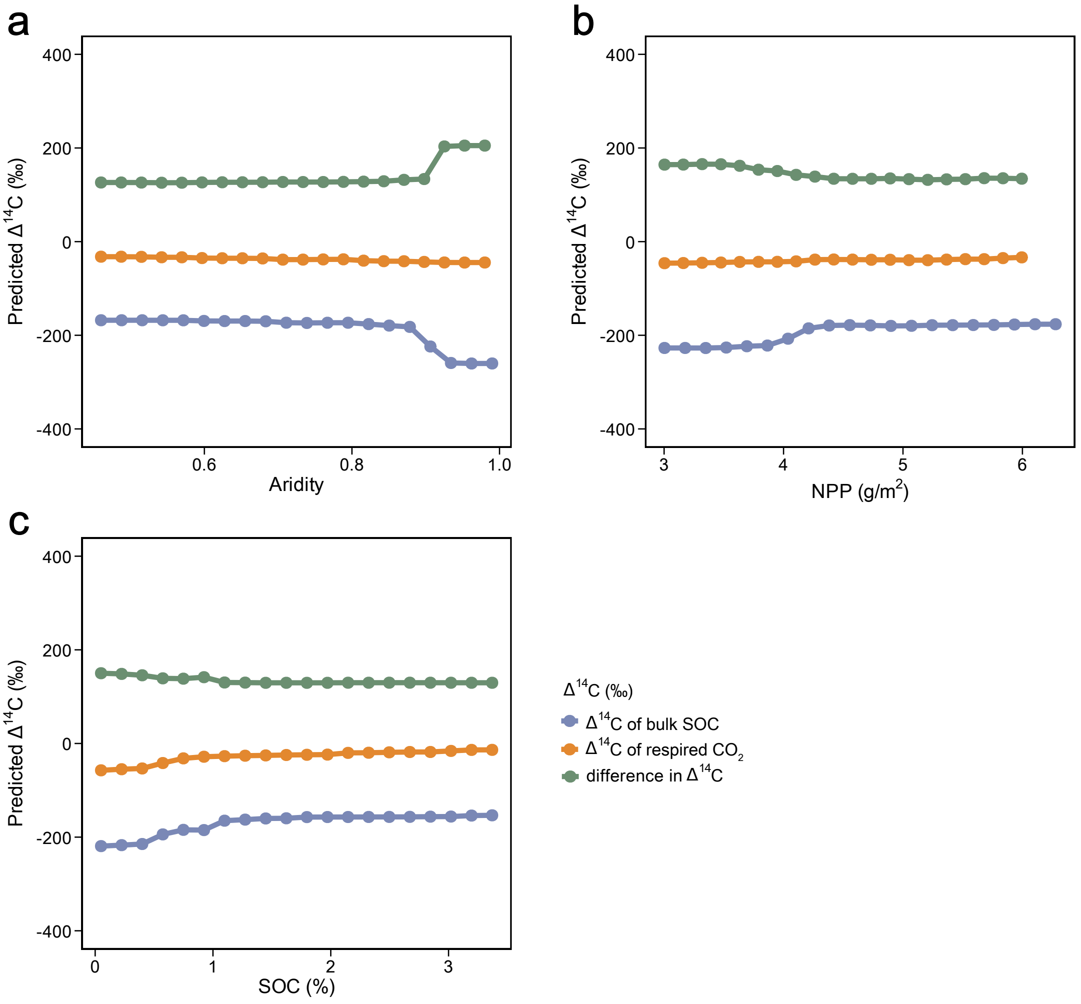


**Extended Data Fig. 13 | Partial dependence plots (PDPs) visualizing the dependence of $\Delta^{14}C$ values on main predictors.** (**a**) Relationship between predicted $\Delta^{14}C$ values and aridity (1 – aridity index). (**b**) Relationship between predicted $\Delta^{14}C$ values and NPP. (**c**) Relationship between predicted $\Delta^{14}C$ values and SOC. NPP value is ln-transformed. The $\Delta^{14}C$ values based on PDPs showed similar threshold patterns to those obtained from Fig. 3.

**Extended Data Table 1 | The aridity index of all sites reporting paired $\Delta^{14}C$ of bulk SOC and respired $CO_2$ in global drylands.**

| Aridity index value | Climate class | This study | ISRaD |
|---|---|---|---|
| < 0.03 | Hyper Arid | 2 | NA |
| 0.03 – 0.20 | Arid | 49 | 2 |
| 0.20 – 0.50 | Semi-Arid | 43 | 9 |
| 0.50 – 0.65 | Dry sub-humid | 3 | 2 |

Our dataset significantly enhances the spatial coverage and representativeness of paired $\Delta^{14}C$ measurements of bulk SOC and respired $CO_2$, extending from a limited set of independent sites across five countries on three continents to continuous aridity gradient regions spanning ten countries on six continents. Our paired $\Delta^{14}C$ samples of bulk SOC and respired $CO_2$ represent more than a sevenfold increase over ISRaD, marking a major advancement in both the quantity and spatial scale of dryland soil data.

**Extended Data Table 2 | Estimation of the contribution of fossil OC to bulk SOC.** We estimated the potential fraction of fossil OC in bulk SOC using a two-end-member radiocarbon mass balance. Then we can calculate the fraction of fossil OC ($f_{fossil}$) as follows: $f_{fossil} \times \Delta^{14}C_{fossil} + f_{bio} \times \Delta^{14}C_{bio} = \Delta^{14}C_{SOC}$, $f_{fossil} + f_{bio} = 1$, where $\Delta^{14}C_{fossil}$, $\Delta^{14}C_{bio}$, and $\Delta^{14}C_{SOC}$ represent the $\Delta^{14}C$ of fossil OC (–1000‰), $\Delta^{14}C$ of young OC from recent biospheric inputs (approximated as the $\Delta^{14}C$ of atmosphere $CO_2$ in the sampling year), and $\Delta^{14}C$ of bulk SOC, respectively. $f_{fossil}$ and $f_{bio}$ represent the fractions of fossil carbon and biospheric carbon in SOC. This yields a mean $f_{fossil}$ of 16 ± 1 % (mean ± SE), indicating that fossil OC does not dominate SOC.

| Number | Region | Latitude | Longitude | atm $\Delta^{14}C$ (‰) | bulk $\Delta^{14}C$ (‰) | $f_{fossil}$ |
|---|---|---|---|---|---|---|
| 1 | Argentina | -41.04 | -70.49 | 16.10 | -111.35 | 13% |
| 2 | Argentina | -41.00 | -71.06 | 16.10 | -61.74 | 8% |
| 3 | Argentina | -51.49 | -69.31 | 16.10 | -73.52 | 9% |
| 4 | Argentina | -39.12 | -64.06 | 20.61 | -174.32 | 19% |
| 5 | Argentina | -40.61 | -66.17 | 20.61 | -99.33 | 12% |
| 6 | Argentina | -27.59 | -66.41 | 16.10 | -123.96 | 14% |
| 7 | Argentina | -29.29 | -65.26 | 16.10 | -27.28 | 4% |
| 8 | Argentina | -45.13 | -70.00 | 16.10 | -168.64 | 18% |
| 9 | Argentina | -45.38 | -70.25 | 16.10 | -211.53 | 22% |
| 10 | Argentina | -45.58 | -71.39 | 16.10 | -64.41 | 8% |
| 11 | Australia | -31.08 | 141.70 | 20.61 | -128.26 | 15% |
| 12 | Australia | -34.79 | 144.78 | 20.61 | -103.63 | 12% |
| 13 | Australia | -31.49 | 147.23 | 20.61 | -85.62 | 10% |
| 14 | Australia | -23.36 | 138.54 | 20.61 | -297.32 | 31% |
| 15 | Australia | -23.36 | 138.26 | 20.61 | -305.33 | 32% |
| 16 | Australia | -23.68 | 138.44 | 20.61 | -372.39 | 39% |
| 17 | China_alpine | 31.95 | 93.92 | -2.70 | -68.76 | 7% |
| 18 | China_alpine | 31.67 | 92.34 | -2.70 | -8.56 | 1% |
| 19 | China_alpine | 31.40 | 90.31 | -2.70 | -58.76 | 6% |
| 20 | China_alpine | 31.63 | 89.58 | -2.70 | -30.95 | 3% |
| 21 | China_alpine | 31.49 | 88.91 | -2.70 | -98.77 | 10% |
| 22 | China_alpine | 31.83 | 88.06 | -2.70 | -64.16 | 6% |
| 23 | China_alpine | 31.95 | 86.88 | -2.70 | -100.17 | 10% |
| 24 | China_alpine | 31.92 | 86.46 | -2.70 | -163.50 | 16% |
| 25 | China_alpine | 31.94 | 85.80 | -2.70 | -191.91 | 19% |
| 26 | China_alpine | 32.02 | 85.45 | -2.70 | -280.04 | 28% |
| 27 | China_alpine | 32.31 | 83.76 | -2.70 | -289.94 | 29% |
| 28 | China_alpine | 31.99 | 84.83 | -2.70 | -316.65 | 31% |
| 29 | China_alpine | 32.22 | 84.47 | -2.70 | -388.38 | 39% |
| 30 | China_alpine | 32.25 | 84.27 | -2.70 | -348.06 | 35% |

| | | | | | | |
|---|---|---|---|---|---|---|
| 31 | China_alpine | 32.44 | 83.15 | -2.70 | -347.56 | 35% |
| 32 | China_alpine | 32.42 | 82.81 | -2.70 | -326.76 | 32% |
| 33 | China_alpine | 32.24 | 82.14 | -2.70 | -386.08 | 38% |
| 34 | China_alpine | 32.17 | 81.60 | -2.70 | -281.04 | 28% |
| 35 | China_alpine | 32.27 | 81.25 | -2.70 | -400.38 | 40% |
| 36 | China_alpine | 32.72 | 79.83 | -2.70 | -420.69 | 42% |
| 37 | China_loess | 38.79 | 110.37 | 15.40 | -202.23 | 21% |
| 38 | China_loess | 37.51 | 110.29 | 15.40 | -337.46 | 35% |
| 39 | China_loess | 36.70 | 109.52 | 15.40 | -171.50 | 18% |
| 40 | China_loess | 36.07 | 109.17 | 15.40 | -122.75 | 14% |
| 41 | China_loess | 35.33 | 109.13 | 15.40 | -96.83 | 11% |
| 42 | China_loess | 34.85 | 108.10 | 15.40 | -77.91 | 9% |
| 43 | China_west | 37.87 | 104.99 | -2.70 | -281.81 | 28% |
| 44 | China_west | 36.75 | 109.25 | -2.70 | -81.17 | 8% |
| 45 | China_west | 40.24 | 93.95 | -2.70 | -399.78 | 40% |
| 46 | China_west | 38.60 | 102.97 | -2.70 | -295.14 | 29% |
| 47 | China_west | 37.85 | 107.41 | -2.70 | -122.65 | 12% |
| 48 | China_west | 39.36 | 100.15 | -2.70 | -243.33 | 24% |
| 49 | China_west | 37.90 | 105.21 | -2.70 | -199.38 | 20% |
| 50 | China_west | 37.37 | 107.62 | -2.70 | -209.88 | 21% |
| 51 | China_west | 36.07 | 109.16 | -2.70 | -225.32 | 22% |
| 52 | China_west | 38.25 | 102.43 | -2.70 | -175.80 | 17% |
| 53 | China_west | 43.41 | 88.58 | -2.70 | -157.82 | 16% |
| 54 | China_west | 39.37 | 100.15 | -2.70 | -136.39 | 13% |
| 55 | China_west | 37.65 | 108.78 | -2.70 | -242.83 | 24% |
| 56 | China_west | 38.12 | 109.32 | -2.70 | -146.93 | 14% |
| 57 | China_west | 38.07 | 109.46 | -2.70 | -188.71 | 19% |
| 58 | China_west | 36.65 | 109.27 | -2.70 | -240.22 | 24% |
| 59 | China_west | 36.69 | 109.52 | -2.70 | -198.61 | 20% |
| 60 | China_west | 38.38 | 109.71 | -2.70 | -234.42 | 23% |
| 61 | China_west | 38.58 | 109.69 | -2.70 | -266.33 | 26% |
| 62 | China_east | 39.78 | 110.73 | -2.70 | -242.73 | 24% |
| 63 | China_east | 44.04 | 114.97 | -2.70 | -246.33 | 24% |
| 64 | China_east | 40.97 | 112.28 | -2.70 | -270.44 | 27% |
| 65 | China_east | 41.49 | 113.19 | -2.70 | -252.25 | 25% |
| 66 | China_east | 39.83 | 110.30 | -2.70 | -162.40 | 16% |
| 67 | China_east | 42.74 | 112.69 | -2.70 | -265.43 | 26% |
| 68 | China_east | 43.86 | 113.66 | -2.70 | -182.80 | 18% |
| 69 | China_east | 40.86 | 112.70 | -2.70 | -145.59 | 14% |
| 70 | China_east | 43.13 | 112.92 | -2.70 | -216.12 | 21% |
| 71 | China_east | 43.83 | 113.64 | -2.70 | -167.10 | 16% |
| 72 | China_east | 40.15 | 111.25 | -2.70 | -268.93 | 27% |

| | | | | | | |
|---|---|---|---|---|---|---|
| 73 | Iran | 37.49 | 56.11 | 10.80 | -217.82 | 23% |
| 74 | Iran | 37.51 | 55.88 | 10.80 | -94.34 | 10% |
| 75 | Iran | 36.70 | 58.67 | 10.80 | -130.09 | 14% |
| 76 | Iran | 37.42 | 56.77 | 10.80 | -61.49 | 7% |
| 77 | Iran | 36.60 | 60.39 | 10.80 | -85.12 | 9% |
| 78 | South Africa | -33.17 | 22.27 | 12.74 | -183.01 | 19% |
| 79 | South Africa | -32.62 | 24.69 | 12.74 | -241.55 | 25% |
| 80 | South Africa | -24.14 | 27.29 | 20.64 | -129.06 | 15% |
| 81 | South Africa | -23.83 | 29.69 | 20.64 | -55.39 | 7% |
| 82 | South Africa | -26.08 | 26.21 | 9.25 | -269.16 | 28% |
| 83 | South Africa | -23.83 | 31.21 | 20.64 | -97.73 | 12% |
| 84 | South Africa | -23.14 | 29.61 | 20.64 | -166.29 | 18% |
| 85 | Spain | 39.75 | -5.94 | 10.85 | -66.70 | 8% |
| 86 | Spain | 39.74 | -4.41 | 10.85 | -77.61 | 9% |
| 87 | Spain | 41.40 | -0.72 | 10.85 | -59.59 | 7% |
| 88 | Spain | 41.77 | -0.59 | 10.85 | -43.88 | 5% |
| 89 | Spain | 36.75 | -2.16 | 10.85 | -267.69 | 28% |
| 90 | Spain | 37.12 | -2.50 | 10.85 | -114.94 | 12% |
| 91 | USA | 38.17 | -109.73 | 4.90 | -155.59 | 16% |
| 92 | USA | 36.98 | -111.51 | 4.90 | -349.53 | 35% |
| 93 | USA | 29.48 | -103.92 | 7.40 | -160.71 | 17% |
| 94 | USA | 31.84 | -110.84 | 10.80 | -57.69 | 7% |
| 95 | USA | 31.89 | -110.88 | 10.80 | -98.73 | 11% |
| 96 | USA | 31.79 | -110.82 | 10.80 | -76.91 | 9% |
| 97 | USA | 32.60 | -106.83 | 7.40 | -143.17 | 15% |
| **Mean** | **-** | **-** | **-** | **-** | **-** | **~16%** |

**Extended Data Table 3 | Soil $\Delta^{14}C$ of respired $CO_2$ in global database (include our field sites and ISRaD).** $\Delta^{14}C_{CO2}$, the $\Delta^{14}C$ of respired $CO_2$ (‰, $n$ = 432).

| | Climate | Mean | Median | Minimum | Maximum | *n* |
|---|---|---|---|---|---|---|
| $\Delta^{14}C_{CO2}$ | Hyper Arid | -67.9 | -67.9 | -40.6 | -95.2 | 2 |
| | Arid | -46.3 | -48.4 | 31.5 | -116.8 | 44 |
| | Semi-Arid | 21.4 | 14.3 | 173.7 | -110.7 | 172 |
| | Dry sub-humid | 47.6 | 42.5 | 155.8 | 1.6 | 55 |
| | Sub-humid | 33.2 | 37.2 | 123.5 | -269.2 | 40 |
| | Humid | 48.2 | 47.0 | 140.0 | -32.5 | 119 |

**Extended Data Table 4 | Estimation of the contribution of old SOC to respired $CO_2$ using different assumptions for the age of C in the slow pool.** To estimate the contribution of older C, we used a two-end-member mixing model to quantity the contributions of millennia-old SOC ($f_{old}$) and bomb-derived young C to soil respiration[17]. The proportion of old C in soil respired $CO_2$ ($f_{old}$) was estimated as: $\Delta^{14}C_{CO2} = \Delta^{14}C_{old} \times f_{old} + \Delta^{14}C_{young} \times (1-f_{old})$, where $\Delta^{14}C_{CO2}$ represents the $\Delta^{14}C$ of total $CO_2$ released from the soil (respired $\Delta^{14}C_{sample}$), and $\Delta^{14}C_{young}$ represents $\Delta^{14}C$ of the atmosphere of the sampling year (atm $\Delta^{14}C$). If we assume $\Delta^{14}C_{old}$ represents $\Delta^{14}C$ of bulk SOC (bulk $\Delta^{14}C_{sample}$), the mean $f_{old}$ is 23 ± 3% (mean ± SE), ranging from 6% to 69% across sites. If we assume $\Delta^{14}C_{old}$ to represent $^{14}C$-free petrogenic C (–1000‰), the estimated $f_{old}$ (hereafter $f_{old\text{-}petro}$) is 4 ± 0.4%, ranging from 1% to 12%.

For most of the sites, we have data on the relative amounts of bulk C found in particulate organic C (POC) and mineral-associated organic C (MAOC) fractions; these are often used to approximate fast and slow cycling C fractions[17] and can help make a better approximation of $f_{old.}$ First, we assume that the particulate organic C (mostly assumed to be fast-cycling) will have modern $\Delta^{14}C$ values, and as we know the fraction of bulk OC in POC and MAOC, we can estimate the $\Delta^{14}C_{MAOC}$ using mass balance: bulk $\Delta^{14}C = \Delta^{14}C_{POC} \times w_{POC} + \Delta^{14}C_{MAOC} \times w_{MAOC}$, where $\Delta^{14}C_{POC}$ is assumed to reflect modern C (≈ 0‰), and $w_{POC}$ and $w_{MAOC}$ represent the fractions of POC and MAOC to SOC, respectively. Under these assumptions, the estimated $f_{old,\ MAOC}$ averages 14 ± 2%, ranging from 3% to 53%.

| Number | atm $\Delta^{14}C$ (‰) | bulk $\Delta^{14}C$ (‰) | respired $\Delta^{14}C$ (‰) | $f_{old}$ | $f_{old,\ petro}$ | $f_{old,\ MAOC}$ |
|---|---|---|---|---|---|---|
| 1 | 16.10 | -111.35 | 21.96 | 0% | 0% | 0% |
| 2 | 16.10 | -61.74 | 44.28 | 0% | 0% | 0% |
| 3 | 16.10 | -73.52 | 23.37 | 0% | 0% | 0% |
| 4 | 20.61 | -174.32 | -29.56 | 26% | 5% | 15% |
| 5 | 20.61 | -99.33 | 11.28 | 8% | 1% | 4% |
| 6 | 16.10 | -123.96 | -27.48 | 31% | 4% | 19% |
| 7 | 16.10 | -27.28 | -4.76 | 48% | 2% | 30% |
| 8 | 16.10 | -168.64 | -14.47 | 17% | 3% | 11% |
| 9 | 16.10 | -211.53 | -70.21 | 38% | 8% | 27% |
| 10 | 16.10 | -64.41 | 10.15 | 7% | 1% | 6% |
| 11 | 20.61 | -128.26 | -17.05 | 25% | 4% | 17% |
| 12 | 20.61 | -103.63 | -25.96 | 37% | 5% | 25% |
| 13 | 20.61 | -85.62 | -31.46 | 49% | 5% | 30% |
| 14 | 20.61 | -297.32 | -52.98 | 23% | 7% | 12% |
| 15 | 20.61 | -305.33 | -87.82 | 33% | 11% | 19% |
| 16 | 20.61 | -372.39 | -57.79 | 20% | 8% | 13% |
| 17 | -2.70 | -68.76 | 55.58 | 0% | 0% | NA |

| | | | | | | |
|---|---|---|---|---|---|---|
| 18 | -2.70 | -8.56 | 56.67 | 0% | 0% | NA |
| 19 | -2.70 | -58.76 | 9.57 | 0% | 0% | NA |
| 20 | -2.70 | -30.95 | 53.66 | 0% | 0% | NA |
| 21 | -2.70 | -98.77 | NA | NA | NA | NA |
| 22 | -2.70 | -64.16 | 6.77 | 0% | 0% | NA |
| 23 | -2.70 | -100.17 | -12.96 | 11% | 1% | NA |
| 24 | -2.70 | -163.50 | -23.48 | 13% | 2% | NA |
| 25 | -2.70 | -191.91 | NA | NA | NA | NA |
| 26 | -2.70 | -280.04 | NA | NA | NA | NA |
| 27 | -2.70 | -289.94 | -59.02 | 20% | 6% | NA |
| 28 | -2.70 | -316.65 | NA | NA | NA | NA |
| 29 | -2.70 | -388.38 | NA | NA | NA | NA |
| 30 | -2.70 | -348.06 | -97.02 | 27% | 9% | NA |
| 31 | -2.70 | -347.56 | NA | NA | NA | NA |
| 32 | -2.70 | -326.76 | -97.94 | 29% | 10% | NA |
| 33 | -2.70 | -386.08 | NA | NA | NA | NA |
| 34 | -2.70 | -281.04 | NA | NA | NA | NA |
| 35 | -2.70 | -400.38 | -119.51 | 29% | 12% | NA |
| 36 | -2.70 | -420.69 | -90.87 | 21% | 9% | NA |
| 37 | 15.40 | -202.23 | NA | NA | NA | NA |
| 38 | 15.40 | -337.46 | NA | NA | NA | NA |
| 39 | 15.40 | -171.50 | NA | NA | NA | NA |
| 40 | 15.40 | -122.75 | NA | NA | NA | NA |
| 41 | 15.40 | -96.83 | NA | NA | NA | NA |
| 42 | 15.40 | -77.91 | NA | NA | NA | NA |
| 43 | -2.70 | -281.81 | -103.67 | 36% | 10% | NA |
| 44 | -2.70 | -81.17 | -10.45 | 10% | 1% | NA |
| 45 | -2.70 | -399.78 | NA | NA | NA | NA |
| 46 | -2.70 | -295.14 | -80.87 | 27% | 8% | NA |
| 47 | -2.70 | -122.65 | -44.45 | 35% | 4% | NA |
| 48 | -2.70 | -243.33 | -87.27 | 35% | 8% | NA |
| 49 | -2.70 | -199.38 | -83.09 | 41% | 8% | NA |
| 50 | -2.70 | -209.88 | NA | NA | NA | NA |
| 51 | -2.70 | -225.32 | -48.71 | 21% | 5% | NA |
| 52 | -2.70 | -175.80 | -77.44 | 43% | 7% | NA |
| 53 | -2.70 | -157.82 | -62.09 | 38% | 6% | NA |
| 54 | -2.70 | -136.39 | -62.33 | 45% | 6% | NA |
| 55 | -2.70 | -242.83 | -83.50 | 34% | 8% | NA |
| 56 | -2.70 | -146.93 | -80.47 | 54% | 8% | NA |
| 57 | -2.70 | -188.71 | -78.37 | 41% | 8% | NA |
| 58 | -2.70 | -240.22 | -44.29 | 18% | 4% | NA |
| 59 | -2.70 | -198.61 | -52.08 | 25% | 5% | NA |

| | | | | | | |
|---|---|---|---|---|---|---|
| 60 | -2.70 | -234.42 | -87.77 | 37% | 9% | NA |
| 61 | -2.70 | -266.33 | -79.37 | 29% | 8% | NA |
| 62 | -2.70 | -242.73 | -30.05 | 11% | 3% | NA |
| 63 | -2.70 | -246.33 | -98.27 | 39% | 10% | NA |
| 64 | -2.70 | -270.44 | -39.75 | 14% | 4% | NA |
| 65 | -2.70 | -252.25 | -20.50 | 7% | 2% | NA |
| 66 | -2.70 | -162.40 | -104.58 | 64% | 10% | NA |
| 67 | -2.70 | -265.43 | -66.00 | 24% | 6% | NA |
| 68 | -2.70 | -182.80 | -35.24 | 18% | 3% | NA |
| 69 | -2.70 | -145.59 | -13.37 | 7% | 1% | NA |
| 70 | -2.70 | -216.12 | -72.60 | 33% | 7% | NA |
| 71 | -2.70 | -167.10 | -47.05 | 27% | 4% | NA |
| 72 | -2.70 | -268.93 | -49.65 | 18% | 5% | NA |
| 73 | 10.80 | -217.82 | NA | NA | NA | NA |
| 74 | 10.80 | -94.34 | 42.34 | 0% | 0% | 0% |
| 75 | 10.80 | -130.09 | -8.74 | 14% | 2% | 8% |
| 76 | 10.80 | -61.49 | 22.89 | 0% | 0% | 0% |
| 77 | 10.80 | -85.12 | 1.27 | 10% | 1% | 5% |
| 78 | 12.74 | -183.01 | -37.00 | 25% | 5% | 16% |
| 79 | 12.74 | -241.55 | -48.31 | 24% | 6% | 14% |
| 80 | 20.64 | -129.06 | 9.58 | 7% | 1% | 6% |
| 81 | 20.64 | -55.39 | -26.16 | 62% | 5% | 53% |
| 82 | 9.25 | -269.16 | -88.35 | 35% | 10% | 25% |
| 83 | 20.64 | -97.73 | -2.44 | 19% | 2% | 15% |
| 84 | 20.64 | -166.29 | -90.02 | 59% | 11% | 46% |
| 85 | 10.85 | -66.70 | 39.81 | 0% | 0% | 0% |
| 86 | 10.85 | -77.61 | 23.99 | 0% | 0% | 0% |
| 87 | 10.85 | -59.59 | 31.20 | 0% | 0% | 0% |
| 88 | 10.85 | -43.88 | 34.10 | 0% | 0% | 0% |
| 89 | 10.85 | -267.69 | -29.76 | 15% | 4% | 13% |
| 90 | 10.85 | -114.94 | 2.97 | 6% | 1% | 3% |
| 91 | 4.90 | -155.59 | 4.63 | 0% | 0% | 0% |
| 92 | 4.90 | -349.53 | -37.10 | 12% | 4% | 6% |
| 93 | 7.40 | -160.71 | -63.81 | 42% | 7% | 27% |
| 94 | 10.80 | -57.69 | -36.17 | 69% | 5% | 48% |
| 95 | 10.80 | -98.73 | -14.75 | 23% | 3% | 16% |
| 96 | 10.80 | -76.91 | -3.14 | 16% | 1% | 12% |
| 97 | 7.40 | -143.17 | -27.58 | 23% | 3% | 19% |
| **Mean** | **-** | **-** | **-** | **~23%** | **~4%** | **~14%** |

**Extended Data Table 5 | Mean age (years) of bulk SOC and respired $CO_2$ in drylands estimated using one-pool models.** The mean ages of bulk SOC and respired $CO_2$ were calculated from $\Delta^{14}C_{sample}$ using one-pool steady-state models fitted to the $\Delta^{14}C$ of bulk SOC and respired $CO_2$. The mean age of bulk SOC is 2100 years (range: 328–6028 years). The mean age of respired $CO_2$ is 520 years (range: 111–1204 years).

| Number | Region | Latitude | Longitude | Mean age of bulk SOC | Mean age of respired $CO_2$ |
|---|---|---|---|---|---|
| 1 | Argentina | -41.04 | -70.49 | 1122 | 199 |
| 2 | Argentina | -41.00 | -71.06 | 680 | 136 |
| 3 | Argentina | -51.49 | -69.31 | 776 | 194 |
| 4 | Argentina | -39.12 | -64.06 | 1802 | 448 |
| 5 | Argentina | -40.61 | -66.17 | 1007 | 237 |
| 6 | Argentina | -27.59 | -66.41 | 1248 | 435 |
| 7 | Argentina | -29.29 | -65.26 | 433 | 308 |
| 8 | Argentina | -45.13 | -70.00 | 1735 | 358 |
| 9 | Argentina | -45.38 | -70.25 | 2264 | 749 |
| 10 | Argentina | -45.58 | -71.39 | 701 | 242 |
| 11 | Australia | -31.08 | 141.70 | 1292 | 372 |
| 12 | Australia | -34.79 | 144.78 | 1048 | 425 |
| 13 | Australia | -31.49 | 147.23 | 881 | 460 |
| 14 | Australia | -23.36 | 138.54 | 3531 | 612 |
| 15 | Australia | -23.36 | 138.26 | 3666 | 901 |
| 16 | Australia | -23.68 | 138.44 | 4932 | 648 |
| 17 | China_alpine | 31.95 | 93.92 | 738 | 113 |
| 18 | China_alpine | 31.67 | 92.34 | 328 | 111 |
| 19 | China_alpine | 31.40 | 90.31 | 657 | 245 |
| 20 | China_alpine | 31.63 | 89.58 | 458 | 117 |
| 21 | China_alpine | 31.49 | 88.91 | 1002 | NA |
| 22 | China_alpine | 31.83 | 88.06 | 700 | 257 |
| 23 | China_alpine | 31.95 | 86.88 | 1015 | 351 |
| 24 | China_alpine | 31.92 | 86.46 | 1676 | 411 |
| 25 | China_alpine | 31.94 | 85.80 | 2015 | NA |
| 26 | China_alpine | 32.02 | 85.45 | 3250 | NA |
| 27 | China_alpine | 32.31 | 83.76 | 3409 | 659 |
| 28 | China_alpine | 31.99 | 84.83 | 3862 | NA |
| 29 | China_alpine | 32.22 | 84.47 | 5276 | NA |
| 30 | China_alpine | 32.25 | 84.27 | 4442 | 986 |
| 31 | China_alpine | 32.44 | 83.15 | 4432 | NA |
| 32 | China_alpine | 32.42 | 82.81 | 4042 | 994 |
| 33 | China_alpine | 32.24 | 82.14 | 5225 | NA |
| 34 | China_alpine | 32.17 | 81.60 | 3266 | NA |

| | | | | | |
|---|---|---|---|---|---|
| 35 | China_alpine | 32.27 | 81.25 | 5546 | 1204 |
| 36 | China_alpine | 32.72 | 79.83 | 6028 | 929 |
| 37 | China_loess | 38.79 | 110.37 | 2144 | NA |
| 38 | China_loess | 37.51 | 110.29 | 4240 | NA |
| 39 | China_loess | 36.70 | 109.52 | 1769 | NA |
| 40 | China_loess | 36.07 | 109.17 | 1236 | NA |
| 41 | China_loess | 35.33 | 109.13 | 984 | NA |
| 42 | China_loess | 34.85 | 108.10 | 815 | NA |
| 43 | China_west | 37.87 | 104.99 | 3278 | 1049 |
| 44 | China_west | 36.75 | 109.25 | 843 | 338 |
| 45 | China_west | 40.24 | 93.95 | 5532 | NA |
| 46 | China_west | 38.60 | 102.97 | 3495 | 840 |
| 47 | China_west | 37.85 | 107.41 | 1235 | 550 |
| 48 | China_west | 39.36 | 100.15 | 2699 | 897 |
| 49 | China_west | 37.90 | 105.21 | 2108 | 860 |
| 50 | China_west | 37.37 | 107.62 | 2243 | NA |
| 51 | China_west | 36.07 | 109.16 | 2448 | 581 |
| 52 | China_west | 38.25 | 102.43 | 1819 | 811 |
| 53 | China_west | 43.41 | 88.58 | 1612 | 683 |
| 54 | China_west | 39.37 | 100.15 | 1378 | 685 |
| 55 | China_west | 37.65 | 108.78 | 2691 | 863 |
| 56 | China_west | 38.12 | 109.32 | 1491 | 837 |
| 57 | China_west | 38.07 | 109.46 | 1975 | 819 |
| 58 | China_west | 36.65 | 109.27 | 2655 | 549 |
| 59 | China_west | 36.69 | 109.52 | 2098 | 606 |
| 60 | China_west | 38.38 | 109.71 | 2573 | 901 |
| 61 | China_west | 38.58 | 109.69 | 3038 | 827 |
| 62 | China_east | 39.78 | 110.73 | 2690 | 452 |
| 63 | China_east | 44.04 | 114.97 | 2742 | 998 |
| 64 | China_east | 40.97 | 112.28 | 3100 | 517 |
| 65 | China_east | 41.49 | 113.19 | 2828 | 394 |
| 66 | China_east | 39.83 | 110.30 | 1664 | 1057 |
| 67 | China_east | 42.74 | 112.69 | 3024 | 715 |
| 68 | China_east | 43.86 | 113.66 | 1903 | 486 |
| 69 | China_east | 40.86 | 112.70 | 1476 | 353 |
| 70 | China_east | 43.13 | 112.92 | 2325 | 770 |
| 71 | China_east | 43.83 | 113.64 | 1718 | 569 |
| 72 | China_east | 40.15 | 111.25 | 3077 | 588 |
| 73 | Iran | 37.49 | 56.11 | 2347 | NA |
| 74 | Iran | 37.51 | 55.88 | 961 | 142 |
| 75 | Iran | 36.70 | 58.67 | 1312 | 329 |
| 76 | Iran | 37.42 | 56.77 | 679 | 197 |

| | | | | | |
|---|---|---|---|---|---|
| 77 | Iran | 36.60 | 60.39 | 878 | 281 |
| 78 | South Africa | -33.17 | 22.27 | 1906 | 497 |
| 79 | South Africa | -32.62 | 24.69 | 2673 | 577 |
| 80 | South Africa | -24.14 | 27.29 | 1301 | 244 |
| 81 | South Africa | -23.83 | 29.69 | 630 | 426 |
| 82 | South Africa | -26.08 | 26.21 | 3081 | 906 |
| 83 | South Africa | -23.83 | 31.21 | 992 | 296 |
| 84 | South Africa | -23.14 | 29.61 | 1708 | 921 |
| 85 | Spain | 39.75 | -5.94 | 721 | 148 |
| 86 | Spain | 39.74 | -4.41 | 812 | 193 |
| 87 | Spain | 41.40 | -0.72 | 664 | 171 |
| 88 | Spain | 41.77 | -0.59 | 546 | 163 |
| 89 | Spain | 36.75 | -2.16 | 3058 | 450 |
| 90 | Spain | 37.12 | -2.50 | 1158 | 273 |
| 91 | USA | 38.17 | -109.73 | 1587 | 266 |
| 92 | USA | 36.98 | -111.51 | 4471 | 499 |
| 93 | USA | 29.48 | -103.92 | 1644 | 697 |
| 94 | USA | 31.84 | -110.84 | 649 | 492 |
| 95 | USA | 31.89 | -110.88 | 1002 | 361 |
| 96 | USA | 31.79 | -110.82 | 806 | 301 |
| 97 | USA | 32.60 | -106.83 | 1450 | 436 |
| **Mean** | **-** | **-** | **-** | **~2100** | **~520** |

**Extended Data Table 6 | Mean transit time ($\tau$) of dryland soils estimated using three different approaches.** $\tau_{CO2}$: based on measured $\Delta^{14}C$ values and assuming a one-pool model (see Statistical analysis). $\tau_{fold}$: based on estimated contributions of old, slow-cycling C pool and its age estimates and assuming a one-pool model (see Supplementary text). $\tau_{machine}$: based on global machine learning-based dataset developed by Zhang et al.[18], where transit time ($\tau_{soil}$) is defined as the ratio of SOC stock to C input or output flux. NA values in $\tau_{CO2}$ and $\tau_{fold}$ mean 17 excluded sites due to uncertainty in $\Delta^{14}C$ of respired $CO_2$. NA values in $\tau_{machine}$ mean missing predictions in the global database.

| Number | $\tau_{CO2}$ (yr) | $\tau_{fold}$ (yr) | $\tau_{machine}$ (yr) |
|---|---|---|---|
| 1 | 199 | 0 | 36 |
| 2 | 136 | 0 | 31 |
| 3 | 194 | 0 | 39 |
| 4 | 448 | 464 | 14 |
| 5 | 237 | 78 | 36 |
| 6 | 435 | 388 | 26 |
| 7 | 308 | 208 | 20 |
| 8 | 358 | 287 | 54 |
| 9 | 749 | 858 | 41 |
| 10 | 242 | 52 | 28 |
| 11 | 372 | 327 | 38 |
| 12 | 425 | 393 | 11 |
| 13 | 460 | 432 | 4 |
| 14 | 612 | 817 | 64 |
| 15 | 901 | 1220 | 50 |
| 16 | 648 | 984 | 85 |
| 17 | 113 | 0 | 77 |
| 18 | 111 | 0 | 76 |
| 19 | 245 | 0 | 35 |
| 20 | 117 | 0 | 47 |
| 21 | NA | NA | 62 |
| 22 | 257 | 0 | 74 |
| 23 | 351 | 107 | 79 |
| 24 | 411 | 217 | 78 |
| 25 | NA | NA | NA |
| 26 | NA | NA | NA |
| 27 | 659 | 668 | NA |
| 28 | NA | NA | NA |
| 29 | NA | NA | 74 |
| 30 | 986 | 1213 | 72 |

| | | | |
|---|---|---|---|
| 31 | NA | NA | 83 |
| 32 | 994 | 1188 | NA |
| 33 | NA | NA | NA |
| 34 | NA | NA | 83 |
| 35 | 1204 | 1629 | NA |
| 36 | 929 | 1272 | NA |
| 37 | NA | NA | 60 |
| 38 | NA | NA | 18 |
| 39 | NA | NA | 35 |
| 40 | NA | NA | 26 |
| 41 | NA | NA | 25 |
| 42 | NA | NA | 24 |
| 43 | 1049 | 1186 | NA |
| 44 | 338 | 83 | 21 |
| 45 | NA | NA | NA |
| 46 | 840 | 934 | 31 |
| 47 | 550 | 430 | 12 |
| 48 | 897 | 949 | NA |
| 49 | 860 | 862 | NA |
| 50 | NA | NA | 18 |
| 51 | 581 | 506 | 26 |
| 52 | 811 | 785 | NA |
| 53 | 683 | 617 | 40 |
| 54 | 685 | 615 | NA |
| 55 | 863 | 905 | 22 |
| 56 | 837 | 804 | 27 |
| 57 | 819 | 803 | 21 |
| 58 | 549 | 465 | 50 |
| 59 | 606 | 529 | 35 |
| 60 | 901 | 945 | 34 |
| 61 | 827 | 884 | 33 |
| 62 | 452 | 307 | 19 |
| 63 | 998 | 1076 | 68 |
| 64 | 517 | 429 | 29 |
| 65 | 394 | 202 | 20 |
| 66 | 1057 | 1062 | 21 |
| 67 | 715 | 729 | 30 |
| 68 | 486 | 344 | 59 |
| 69 | 353 | 110 | 39 |
| 70 | 770 | 761 | 53 |
| 71 | 569 | 463 | 60 |
| 72 | 588 | 543 | 19 |

| | | | |
|---|---|---|---|
| 73 | NA | NA | 23 |
| 74 | 142 | 0 | 30 |
| 75 | 329 | 182 | 40 |
| 76 | 197 | 0 | 30 |
| 77 | 281 | 87 | 19 |
| 78 | 497 | 484 | 28 |
| 79 | 577 | 642 | 29 |
| 80 | 244 | 96 | 22 |
| 81 | 426 | 388 | 18 |
| 82 | 906 | 1080 | 18 |
| 83 | 296 | 193 | 13 |
| 84 | 921 | 1011 | 24 |
| 85 | 148 | 0 | 26 |
| 86 | 193 | 0 | 21 |
| 87 | 171 | 0 | 41 |
| 88 | 163 | 0 | 15 |
| 89 | 450 | 446 | 25 |
| 90 | 273 | 73 | 33 |
| 91 | 266 | 3 | 21 |
| 92 | 499 | 530 | NA |
| 93 | 697 | 696 | 30 |
| 94 | 492 | 445 | 26 |
| 95 | 361 | 234 | 26 |
| 96 | 301 | 128 | 30 |
| 97 | 436 | 337 | 30 |
| **Mean** | **~520** | **~480** | **~37** |

**Extended Data Table 7 | Comparisons of the results from one-pool and two-pool models.** (**a**) Transit time vs. bulk age. (**b**) Transit time of fast pool vs. slow pool. $1P_{bulk}$, one-pool model results based on $\Delta^{14}C$ of bulk SOC; $1P_{inc}$, one-pool model results based on $\Delta^{14}C$ of respired $CO_2$; $2P_{Parallel}$, two-pool parallel model results; $2P_{Series}$, two-pool series model results. Overall refers to the model results from two-pool parallel models in all dryland sites. Values in parentheses indicate the 95% confidence interval (lower and upper bounds).

(**a**)

| | Model | Transit time | | Bulk age | |
|---|---|---|---|---|---|
| | | **Median (yr)** | **Mean (yr)** | **Median (yr)** | **Mean (yr)** |
| Arid | $1P_{bulk}$ | - | - | - | 2583 |
| | $1P_{inc}$ | - | 490 | - | - |
| | $2P_{Parallel}$ | 3.0 | 540 | 1194 | 1726 |
| | $2P_{Series}$ | 3.8 | 247 | 1507 | 2192 |
| Semi-Arid | $1P_{bulk}$ | - | - | - | 1445 |
| | $1P_{inc}$ | - | 346 | - | - |
| | $2P_{Parallel}$ | 71.0 | 356 | 42530 | 62140 |
| | $2P_{Series}$ | 29.6 | 212 | 315 | 417 |
| Overall | $2P_{Parallel}$ | 8.5 | 253 | 347 | 502 |

(**b**)

| | Model | Transit time of fast pool | | | Transit time of slow pool | | |
|---|---|---|---|---|---|---|---|
| | | **Mean (yr)** | **Mass (%)** | **Flux (%)** | **Mean (yr)** | **Mass (%)** | **Flux (%)** |
| Arid | $1P_{bulk}$ | - | - | - | 2583 (1390,4140) | - | - |
| | $1P_{inc}$ | 490 (115,1334) | - | - | - | - | - |
| | $2P_{Parallel}$ | 2.3 (1.5,5.2) | 0.3 | 68.9 | 1724 (1563,2564) | 99.7 | 31.1 |
| | $2P_{Series}$ | 4.7 (2.7,17.7) | 1.9 | 90.1 | 2222 (1380,5695) | 98.1 | 9.9 |
| Semi-Arid | $1P_{bulk}$ | - | - | - | 1445 (697,2583) | - | - |
| | $1P_{inc}$ | 346 (204,1163) | - | - | - | - | - |
| | $2P_{Parallel}$ | 19.7 (8.9,38.5) | 2.5 | 45.5 | 637 (290,1176) | 97.5 | 54.5 |
| | $2P_{Series}$ | 18.0 (17.6,39.7) | 8.3 | 71.2 | 500 (188,1250) | 91.7 | 28.8 |
| Overall | $2P_{Parallel}$ | 2.1(1.4,3.9) | 0.4 | 49.9 | 505 (204,1124) | 99.6 | 50.1 |

**Extended Data Table 8 | Global dryland site information including site location, climate variables, vegetation factors, soil properties, and SIC contribution to $CO_2$ efflux ($f_{SIC}$).**

**(a)** Site location.

| Number | Region | Latitude (°) | Longitude (°) | Sampling year | Grazing |
|---|---|---|---|---|---|
| 1 | Argentina | -41.04 | -70.49 | 2017 | no |
| 2 | Argentina | -41.00 | -71.06 | 2017 | low |
| 3 | Argentina | -51.49 | -69.31 | 2017 | low |
| 4 | Argentina | -39.12 | -64.06 | 2016 | low |
| 5 | Argentina | -40.61 | -66.17 | 2016 | low |
| 6 | Argentina | -27.59 | -66.41 | 2017 | low |
| 7 | Argentina | -29.29 | -65.26 | 2017 | low |
| 8 | Argentina | -45.13 | -70.00 | 2017 | no |
| 9 | Argentina | -45.38 | -70.25 | 2017 | no |
| 10 | Argentina | -45.58 | -71.39 | 2017 | no |
| 11 | Australia | -31.08 | 141.70 | 2016 | no |
| 12 | Australia | -34.79 | 144.78 | 2016 | no |
| 13 | Australia | -31.49 | 147.23 | 2016 | low |
| 14 | Australia | -23.36 | 138.54 | 2016 | no |
| 15 | Australia | -23.36 | 138.26 | 2016 | no |
| 16 | Australia | -23.68 | 138.44 | 2016 | no |
| 17 | China_alpine | 31.95 | 93.92 | 2020 | no |
| 18 | China_alpine | 31.67 | 92.34 | 2020 | no |
| 19 | China_alpine | 31.40 | 90.31 | 2020 | no |
| 20 | China_alpine | 31.63 | 89.58 | 2020 | no |
| 21 | China_alpine | 31.49 | 88.91 | 2020 | cold-season |
| 22 | China_alpine | 31.83 | 88.06 | 2020 | cold-season |
| 23 | China_alpine | 31.95 | 86.88 | 2020 | no |
| 24 | China_alpine | 31.92 | 86.46 | 2020 | cold-season |
| 25 | China_alpine | 31.94 | 85.80 | 2020 | cold-season |
| 26 | China_alpine | 32.02 | 85.45 | 2020 | cold-season |
| 27 | China_alpine | 32.31 | 83.76 | 2020 | cold-season |
| 28 | China_alpine | 31.99 | 84.83 | 2020 | no |
| 29 | China_alpine | 32.22 | 84.47 | 2020 | cold-season |
| 30 | China_alpine | 32.25 | 84.27 | 2020 | cold-season |
| 31 | China_alpine | 32.44 | 83.15 | 2020 | cold-season |
| 32 | China_alpine | 32.42 | 82.81 | 2020 | cold-season |
| 33 | China_alpine | 32.24 | 82.14 | 2020 | no |
| 34 | China_alpine | 32.17 | 81.60 | 2020 | no |

| | | | | | |
|---|---|---|---|---|---|
| 35 | China_alpine | 32.27 | 81.25 | 2020 | cold-season |
| 36 | China_alpine | 32.72 | 79.83 | 2020 | no |
| 37 | China_loess | 38.79 | 110.37 | 2015 | no |
| 38 | China_loess | 37.51 | 110.29 | 2015 | no |
| 39 | China_loess | 36.70 | 109.52 | 2015 | no |
| 40 | China_loess | 36.07 | 109.17 | 2015 | no |
| 41 | China_loess | 35.33 | 109.13 | 2015 | no |
| 42 | China_loess | 34.85 | 108.10 | 2015 | no |
| 43 | China_west | 37.87 | 104.99 | 2020 | no |
| 44 | China_west | 36.75 | 109.25 | 2020 | no |
| 45 | China_west | 40.24 | 93.95 | 2020 | no |
| 46 | China_west | 38.60 | 102.97 | 2020 | no |
| 47 | China_west | 37.85 | 107.41 | 2020 | no |
| 48 | China_west | 39.36 | 100.15 | 2020 | no |
| 49 | China_west | 37.90 | 105.21 | 2020 | no |
| 50 | China_west | 37.37 | 107.62 | 2020 | no |
| 51 | China_west | 36.07 | 109.16 | 2020 | no |
| 52 | China_west | 38.25 | 102.43 | 2020 | no |
| 53 | China_west | 43.41 | 88.58 | 2020 | no |
| 54 | China_west | 39.37 | 100.15 | 2020 | no |
| 55 | China_west | 37.65 | 108.78 | 2020 | no |
| 56 | China_west | 38.12 | 109.32 | 2020 | no |
| 57 | China_west | 38.07 | 109.46 | 2020 | no |
| 58 | China_west | 36.65 | 109.27 | 2020 | low |
| 59 | China_west | 36.69 | 109.52 | 2020 | no |
| 60 | China_west | 38.38 | 109.71 | 2020 | no |
| 61 | China_west | 38.58 | 109.69 | 2020 | no |
| 62 | China_east | 39.78 | 110.73 | 2020 | low |
| 63 | China_east | 44.04 | 114.97 | 2020 | low |
| 64 | China_east | 40.97 | 112.28 | 2020 | no |
| 65 | China_east | 41.49 | 113.19 | 2020 | low |
| 66 | China_east | 39.83 | 110.30 | 2020 | no |
| 67 | China_east | 42.74 | 112.69 | 2020 | no |
| 68 | China_east | 43.86 | 113.66 | 2020 | low |
| 69 | China_east | 40.86 | 112.70 | 2020 | low |
| 70 | China_east | 43.13 | 112.92 | 2020 | low |
| 71 | China_east | 43.83 | 113.64 | 2020 | low |
| 72 | China_east | 40.15 | 111.25 | 2020 | no |
| 73 | Iran | 37.49 | 56.11 | 2016 | low |
| 74 | Iran | 37.51 | 55.88 | 2016 | low |
| 75 | Iran | 36.70 | 58.67 | 2016 | no |
| 76 | Iran | 37.42 | 56.77 | 2016 | no |

| | | | | | |
|---|---|---|---|---|---|
| 77 | Iran | 36.60 | 60.39 | 2016 | no |
| 78 | South Africa | -33.17 | 22.27 | 2018 | no |
| 79 | South Africa | -32.62 | 24.69 | 2018 | no |
| 80 | South Africa | -24.14 | 27.29 | 2016 | no |
| 81 | South Africa | -23.83 | 29.69 | 2016 | no |
| 82 | South Africa | -26.08 | 26.21 | 2019 | no |
| 83 | South Africa | -23.83 | 31.21 | 2016 | low |
| 84 | South Africa | -23.14 | 29.61 | 2016 | no |
| 85 | Spain | 39.75 | -5.94 | 2016 | no |
| 86 | Spain | 39.74 | -4.41 | 2016 | no |
| 87 | Spain | 41.40 | -0.72 | 2016 | low |
| 88 | Spain | 41.77 | -0.59 | 2016 | low |
| 89 | Spain | 36.75 | -2.16 | 2016 | no |
| 90 | Spain | 37.12 | -2.50 | 2016 | low |
| 91 | USA | 38.17 | -109.73 | 2018 | low |
| 92 | USA | 36.98 | -111.51 | 2018 | no |
| 93 | USA | 29.48 | -103.92 | 2017 | no |
| 94 | USA | 31.84 | -110.84 | 2016 | no |
| 95 | USA | 31.89 | -110.88 | 2016 | no |
| 96 | USA | 31.79 | -110.82 | 2016 | no |
| 97 | USA | 32.60 | -106.83 | 2017 | no |

**(b)** Climate variables.

| Number | Aridity index | Aridity | MAT (°C) |
|---|---|---|---|
| 1 | 0.29 | 0.71 | 8.0 |
| 2 | 0.50 | 0.50 | 7.5 |
| 3 | 0.21 | 0.79 | 7.1 |
| 4 | 0.18 | 0.82 | 15.2 |
| 5 | 0.13 | 0.87 | 14.7 |
| 6 | 0.16 | 0.84 | 19.7 |
| 7 | 0.22 | 0.78 | 20.5 |
| 8 | 0.11 | 0.89 | 9.7 |
| 9 | 0.13 | 0.87 | 9.6 |
| 10 | 0.45 | 0.55 | 7.5 |
| 11 | 0.10 | 0.90 | 19.3 |
| 12 | 0.19 | 0.81 | 17.1 |
| 13 | 0.24 | 0.76 | 19.0 |
| 14 | 0.08 | 0.92 | 24.0 |

| | | | |
|---|---|---|---|
| 15 | 0.08 | 0.92 | 23.7 |
| 16 | 0.07 | 0.93 | 24.0 |
| 17 | 0.54 | 0.46 | 1.2 |
| 18 | 0.43 | 0.57 | -1.3 |
| 19 | 0.36 | 0.64 | -1.3 |
| 20 | 0.32 | 0.68 | -0.9 |
| 21 | 0.29 | 0.71 | -0.4 |
| 22 | 0.24 | 0.76 | -1.4 |
| 23 | 0.14 | 0.86 | -0.2 |
| 24 | 0.12 | 0.88 | -1.3 |
| 25 | 0.10 | 0.90 | -2.7 |
| 26 | 0.08 | 0.92 | -2.1 |
| 27 | 0.06 | 0.94 | 0.2 |
| 28 | 0.08 | 0.92 | -1.2 |
| 29 | 0.06 | 0.94 | 0.0 |
| 30 | 0.06 | 0.94 | -0.6 |
| 31 | 0.05 | 0.95 | -0.9 |
| 32 | 0.05 | 0.95 | -0.3 |
| 33 | 0.07 | 0.93 | -1.2 |
| 34 | 0.09 | 0.91 | -3.4 |
| 35 | 0.08 | 0.92 | -1.4 |
| 36 | 0.07 | 0.93 | -2.7 |
| 37 | 0.28 | 0.72 | 7.4 |
| 38 | 0.17 | 0.83 | 9.6 |
| 39 | 0.36 | 0.64 | 9.5 |
| 40 | 0.40 | 0.60 | 9.7 |
| 41 | 0.46 | 0.54 | 10.2 |
| 42 | 0.53 | 0.47 | 10.1 |
| 43 | 0.12 | 0.88 | 8.5 |
| 44 | 0.36 | 0.64 | 9.0 |
| 45 | 0.01 | 0.99 | 10.2 |
| 46 | 0.08 | 0.92 | 8.0 |
| 47 | 0.21 | 0.79 | 8.0 |
| 48 | 0.10 | 0.90 | 8.1 |
| 49 | 0.12 | 0.88 | 8.5 |
| 50 | 0.27 | 0.73 | 7.2 |
| 51 | 0.41 | 0.59 | 9.3 |
| 52 | 0.11 | 0.89 | 7.6 |
| 53 | 0.13 | 0.87 | 4.3 |
| 54 | 0.10 | 0.90 | 8.1 |
| 55 | 0.28 | 0.72 | 8.2 |
| 56 | 0.25 | 0.75 | 9.0 |

| | | | |
|---|---|---|---|
| 57 | 0.26 | 0.74 | 9.2 |
| 58 | 0.37 | 0.63 | 9.4 |
| 59 | 0.36 | 0.64 | 9.5 |
| 60 | 0.26 | 0.74 | 8.2 |
| 61 | 0.26 | 0.74 | 7.9 |
| 62 | 0.26 | 0.74 | 6.5 |
| 63 | 0.17 | 0.83 | 0.8 |
| 64 | 0.22 | 0.78 | 4.6 |
| 65 | 0.12 | 0.88 | 3.7 |
| 66 | 0.26 | 0.74 | 5.5 |
| 67 | 0.13 | 0.87 | 4.3 |
| 68 | 0.14 | 0.86 | 2.6 |
| 69 | 0.25 | 0.75 | 3.5 |
| 70 | 0.13 | 0.87 | 3.9 |
| 71 | 0.14 | 0.86 | 2.5 |
| 72 | 0.24 | 0.76 | 6.6 |
| 73 | 0.17 | 0.83 | 13.8 |
| 74 | 0.18 | 0.82 | 12.2 |
| 75 | 0.24 | 0.76 | 8.0 |
| 76 | 0.21 | 0.79 | 10.3 |
| 77 | 0.12 | 0.88 | 16.1 |
| 78 | 0.12 | 0.88 | 16.4 |
| 79 | 0.15 | 0.85 | 18.6 |
| 80 | 0.23 | 0.77 | 20.5 |
| 81 | 0.45 | 0.55 | 18.4 |
| 82 | 0.29 | 0.71 | 16.8 |
| 83 | 0.29 | 0.71 | 22.4 |
| 84 | 0.30 | 0.70 | 20.1 |
| 85 | 0.32 | 0.68 | 15.9 |
| 86 | 0.25 | 0.75 | 15.3 |
| 87 | 0.21 | 0.79 | 14.7 |
| 88 | 0.25 | 0.75 | 13.9 |
| 89 | 0.02 | 0.98 | 17.4 |
| 90 | 0.21 | 0.79 | 14.6 |
| 91 | 0.13 | 0.87 | 11.9 |
| 92 | 0.08 | 0.92 | 15.1 |
| 93 | 0.16 | 0.84 | 18.4 |
| 94 | 0.18 | 0.82 | 18.5 |
| 95 | 0.15 | 0.85 | 19.3 |
| 96 | 0.22 | 0.78 | 17.3 |
| 97 | 0.12 | 0.88 | 15.1 |

**(c)** Vegetation factors.

| Number | NPP ($g/m^2$) | Vegetation type | Vegetation classification | Plant cover (%) | Species richness |
|---|---|---|---|---|---|
| 1 | 50.3 | grassland | NA | 29.6 | 26 |
| 2 | 88.6 | grassland | NA | 54.8 | 16 |
| 3 | 164.9 | grassland | NA | 71.8 | 37 |
| 4 | 198.4 | shrubland | NA | 45.3 | 30 |
| 5 | 96.7 | shrubland | NA | 38.0 | 20 |
| 6 | 78.6 | shrubland | NA | 31.6 | 5 |
| 7 | 281.5 | forest | NA | 51.7 | 44 |
| 8 | 231.6 | shrubland | NA | 25.8 | 14 |
| 9 | 51.0 | shrubland | NA | 35.5 | 14 |
| 10 | 121.6 | grassland | NA | 34.4 | 33 |
| 11 | 63.6 | shrubland | NA | 38.7 | 18 |
| 12 | 149.2 | shrubland | NA | 32.4 | 5 |
| 13 | 153.7 | shrubland | NA | 30.2 | 9 |
| 14 | 72.3 | grassland | NA | 44.8 | 14 |
| 15 | 66.5 | grassland | NA | 32.1 | 14 |
| 16 | 70.3 | grassland | NA | 26.2 | 3 |
| 17 | 355.9 | alpine meadow | C3, C4 | 47.0 | 33 |
| 18 | 401.3 | alpine meadow | C3 | 46.7 | 31 |
| 19 | 70.5 | alpine meadow | C3, C4 | 32.7 | 25 |
| 20 | 85.0 | alpine meadow | C3 | 44.4 | 15 |
| 21 | 408.0 | alpine meadow | C3, C4 | 88.2 | 44 |
| 22 | 74.1 | alpine meadow | C3, C4 | 45.4 | 27 |
| 23 | 64.6 | alpine meadow | C3, C4 | 33.3 | 16 |
| 24 | 63.0 | alpine meadow | C3, C4 | 32.3 | 21 |
| 25 | 53.9 | alpine meadow | C3, C4 | 50.2 | 29 |
| 26 | 50.5 | alpine meadow | C3, C4 | 21.6 | 29 |
| 27 | 42.2 | alpine meadow | C3, C4 | 40.2 | 31 |
| 28 | 49.4 | alpine meadow | C3, C4 | 28.6 | 25 |
| 29 | 53.7 | alpine meadow | C3, C4 | 10.6 | 23 |
| 30 | 54.5 | alpine meadow | C3, C4 | 24.4 | 26 |
| 31 | 54.5 | alpine meadow | C3 | 29.7 | 13 |
| 32 | 22.8 | alpine meadow | C3 | 18.9 | 13 |
| 33 | 27.5 | alpine meadow | C3 | 24.0 | 13 |
| 34 | 40.4 | alpine meadow | C3, C4 | 21.4 | 21 |
| 35 | 24.7 | alpine meadow | C3 | 12.7 | 12 |
| 36 | 29.0 | alpine meadow | C3 | 16.0 | 12 |
| 37 | 235.4 | shrubland | C3, C4 | 35.3 | 8 |
| 38 | 46.2 | shrubland | C3, C4 | 40.3 | 10 |

| | | | | | |
|---|---|---|---|---|---|
| 39 | 387.7 | shrubland | C3, C4 | 43.2 | 11 |
| 40 | 449.1 | shrubland | C3, C4 | 48.3 | 11 |
| 41 | 524.6 | shrubland | C3, C4 | 62.5 | 18 |
| 42 | 532.7 | shrubland | C3, C4 | 75.2 | 16 |
| 43 | 20.2 | shrubland | C3, C4 | 28.0 | 14 |
| 44 | 363.2 | grassland | C3, C4 | 82.6 | 86 |
| 45 | 23.5 | desert | C4 | 8.8 | 2 |
| 46 | 57.6 | shrubland | C3, C4 | 16.5 | 14 |
| 47 | 181.0 | grassland | C3, C4 | 38.9 | 34 |
| 48 | 115.3 | shrubland | C3, C4 | 34.5 | 2 |
| 49 | 57.9 | shrubland | C3, C4 | 27.8 | 16 |
| 50 | 193.9 | grassland | C3, C4 | 24.1 | 34 |
| 51 | 20.9 | grassland | C3, C4 | 86.9 | 81 |
| 52 | 69.2 | shrubland | C3, C4 | 13.4 | 18 |
| 53 | 87.8 | desert | C3, C4 | 56.7 | 9 |
| 54 | 147.6 | shrubland | C3 | 42.2 | 14 |
| 55 | 190.0 | shrubland | C4 | 75.0 | 9 |
| 56 | 205.3 | shrubland | C4 | 45.4 | 6 |
| 57 | 276.4 | shrubland | C4 | 54.0 | 4 |
| 58 | 24.2 | grassland | C3 | 47.5 | 23 |
| 59 | 355.7 | grassland | C4 | 54.9 | 28 |
| 60 | 209.7 | shrubland | C4 | 35.7 | 6 |
| 61 | 174.9 | shrubland | C4 | 55.4 | 9 |
| 62 | 64.3 | grassland | C4 | 72.4 | 12 |
| 63 | 172.3 | grassland | C4 | 73.8 | 12 |
| 64 | 281.5 | grassland | C4 | 71.5 | 19 |
| 65 | 143.6 | grassland | C4 | 23.1 | 12 |
| 66 | 153.5 | grassland | C3 | 82.1 | 11 |
| 67 | 133.0 | grassland | C4 | 57.9 | 14 |
| 68 | 89.6 | grassland | C4 | 76.8 | 12 |
| 69 | 266.5 | grassland | C4 | 79.4 | 21 |
| 70 | 96.8 | grassland | C4 | 65.5 | 10 |
| 71 | 120.4 | grassland | C4 | 70.4 | 12 |
| 72 | 152.7 | shrubland | C4 | 73.1 | 9 |
| 73 | 50.6 | shrubland | NA | 36.8 | 9 |
| 74 | 141.7 | shrubland | NA | 84.4 | 42 |
| 75 | 69.5 | forest | NA | 64.8 | 47 |
| 76 | 226.7 | shrubland | NA | 46.3 | 46 |
| 77 | 93.3 | shrubland | NA | 17.5 | 11 |
| 78 | 43.6 | shrubland | NA | 42.2 | 22 |
| 79 | 74.7 | shrubland | NA | 48.9 | 32 |
| 80 | 198.7 | forest | NA | 68.9 | 37 |

| | | | | | |
|---|---|---|---|---|---|
| 81 | 184.3 | grassland | NA | 35.1 | 41 |
| 82 | 114.6 | grassland | NA | 22.8 | 28 |
| 83 | 186.2 | forest | NA | 28.2 | 41 |
| 84 | 180.1 | forest | NA | 59.2 | 27 |
| 85 | 255.2 | forest | NA | 27.2 | 6 |
| 86 | 239.1 | forest | NA | 25.4 | 7 |
| 87 | 88.5 | shrubland | NA | 30.2 | 19 |
| 88 | 189.9 | shrubland | NA | 67.7 | 24 |
| 89 | 136.4 | grassland | NA | 36.7 | 29 |
| 90 | 80.7 | shrubland | NA | 45.5 | 8 |
| 91 | 69.2 | grassland | NA | 19.5 | 20 |
| 92 | 36.4 | shrubland | NA | 21.3 | 15 |
| 93 | 61.5 | shrubland | NA | 22.9 | 18 |
| 94 | 112.3 | grassland | NA | 40.7 | 12 |
| 95 | 92.6 | shrubland | NA | 19.6 | 17 |
| 96 | 126.6 | grassland | NA | 25.9 | 12 |
| 97 | 62.5 | shrubland | NA | 35.8 | 18 |

**(d)** Soil properties.

| Number | SOC (%) | pH | Clay + silt (%) | Fe + Al (mg/g soil) | Respired rate (μg $CO_2$/g soil/day) |
|---|---|---|---|---|---|
| 1 | 0.99 | 6.05 | 18.67 | 5.59 | 81.57 |
| 2 | 1.57 | 6.75 | 33.55 | 8.07 | 92.32 |
| 3 | 2.91 | 6.66 | 35.66 | 4.84 | 157.59 |
| 4 | 1.69 | 6.94 | 32.34 | 2.84 | 139.02 |
| 5 | 0.59 | 7.96 | 17.55 | 3.17 | 41.52 |
| 6 | 0.52 | 6.50 | 35.78 | 2.84 | 42.75 |
| 7 | 1.72 | 5.69 | 37.11 | 2.20 | 67.40 |
| 8 | 0.56 | 7.87 | 28.56 | 4.88 | 40.38 |
| 9 | 0.46 | 7.09 | 8.33 | 4.08 | 37.16 |
| 10 | 1.90 | 6.76 | 37.45 | 7.75 | 117.49 |
| 11 | 0.82 | 7.52 | 46.88 | 2.99 | 89.15 |
| 12 | 1.10 | 6.58 | 31.89 | 2.44 | 115.26 |
| 13 | 1.43 | 6.02 | 62.00 | 3.32 | 159.29 |
| 14 | 0.19 | 5.75 | 4.34 | 1.56 | 13.50 |
| 15 | 0.19 | 6.26 | 4.67 | 1.19 | 19.88 |
| 16 | 0.19 | 5.91 | 5.56 | 1.22 | 20.18 |
| 17 | 2.24 | 6.88 | 42.62 | 4.92 | 34.35 |
| 18 | 3.21 | 6.80 | 22.29 | 3.03 | 38.40 |

| | | | | | |
|---|---|---|---|---|---|
| 19 | 0.98 | 6.86 | 13.48 | 2.22 | 16.44 |
| 20 | 3.37 | 7.64 | 19.03 | 3.89 | 33.12 |
| 21 | 0.81 | 8.57 | 51.01 | 2.44 | 25.07 |
| 22 | 1.23 | 8.31 | 35.66 | 2.59 | 16.50 |
| 23 | 0.85 | 8.45 | 24.58 | 2.65 | 20.49 |
| 24 | 0.70 | 8.77 | 22.66 | 2.14 | 16.76 |
| 25 | 0.88 | 8.91 | 25.81 | 1.70 | 24.26 |
| 26 | 0.63 | 8.66 | 19.73 | 2.19 | 8.21 |
| 27 | 0.70 | 8.64 | 38.44 | 2.02 | 7.52 |
| 28 | 0.60 | 8.65 | 21.27 | 2.27 | 40.94 |
| 29 | 0.33 | 9.22 | 31.07 | 1.75 | 14.74 |
| 30 | 0.48 | 8.59 | 27.54 | 2.09 | 10.52 |
| 31 | 0.63 | 8.60 | 28.59 | 2.58 | 27.37 |
| 32 | 0.56 | 8.57 | 27.41 | 2.79 | 14.47 |
| 33 | 0.43 | 8.32 | 29.47 | 2.27 | 12.56 |
| 34 | 0.92 | 8.16 | 30.08 | 3.17 | 36.79 |
| 35 | 0.29 | 8.55 | 14.00 | 1.59 | 6.63 |
| 36 | 0.39 | 8.68 | 20.08 | 1.57 | 8.25 |
| 37 | 0.39 | 9.02 | 91.62 | 2.35 | 6.97 |
| 38 | 0.40 | 8.81 | 86.87 | 1.92 | 26.04 |
| 39 | 0.56 | 8.85 | 84.22 | 2.48 | 27.57 |
| 40 | 1.19 | 8.42 | 87.15 | 2.54 | 68.57 |
| 41 | 1.28 | 8.24 | 91.88 | 2.44 | 62.11 |
| 42 | 1.47 | 8.15 | 95.64 | 2.51 | 113.48 |
| 43 | 0.13 | 7.78 | 55.50 | 1.92 | 73.23 |
| 44 | 0.89 | 8.79 | 77.38 | 2.32 | 73.59 |
| 45 | 0.06 | 8.28 | 19.12 | 1.60 | 3.41 |
| 46 | 0.13 | 9.76 | 20.57 | 2.13 | 4.85 |
| 47 | 0.50 | 8.48 | 24.54 | 1.42 | 61.86 |
| 48 | 0.05 | 9.39 | 10.72 | 1.84 | 4.88 |
| 49 | 0.22 | 7.84 | 16.06 | 0.89 | 12.70 |
| 50 | 0.39 | 7.87 | 52.12 | 1.98 | 24.48 |
| 51 | 0.92 | 8.17 | 81.85 | 2.63 | 104.44 |
| 52 | 0.19 | 8.67 | 59.23 | 2.13 | 9.10 |
| 53 | 0.44 | 8.70 | 55.50 | 2.66 | 40.96 |
| 54 | 0.49 | 8.30 | 62.50 | 3.96 | 40.08 |
| 55 | 0.20 | 8.19 | 0.68 | 1.15 | 45.33 |
| 56 | 0.16 | 7.91 | 0.21 | 1.16 | 15.62 |
| 57 | 0.16 | 8.15 | 0.59 | 1.17 | 15.83 |
| 58 | 0.79 | 8.38 | 34.83 | 1.96 | 124.89 |
| 59 | 0.09 | 8.92 | 33.81 | 1.89 | 8.48 |
| 60 | 0.14 | 8.50 | 0.59 | 1.13 | 14.58 |

| | | | | | |
|---|---|---|---|---|---|
| 61 | 0.27 | 8.14 | 1.60 | 0.88 | 20.80 |
| 62 | 0.82 | 8.26 | 18.41 | 1.30 | 66.65 |
| 63 | 0.42 | 8.14 | 13.03 | 1.66 | 36.78 |
| 64 | 0.90 | 8.13 | 38.65 | 2.52 | 100.74 |
| 65 | 0.89 | 7.31 | 27.68 | 2.88 | 62.83 |
| 66 | 0.73 | 8.20 | 44.06 | 2.03 | 480.04 |
| 67 | 0.49 | 7.99 | 24.99 | 1.33 | 28.37 |
| 68 | 0.81 | 8.36 | 21.19 | 1.36 | 31.17 |
| 69 | 1.23 | 7.31 | 33.15 | 4.46 | 139.92 |
| 70 | 0.24 | 8.13 | 3.31 | 0.83 | 39.92 |
| 71 | 0.44 | 8.26 | 26.47 | 1.28 | 16.92 |
| 72 | 0.20 | 8.69 | 1.70 | 1.13 | 15.96 |
| 73 | 1.05 | 7.88 | 74.67 | 1.28 | 63.81 |
| 74 | 3.27 | 7.80 | 73.56 | 3.91 | 169.61 |
| 75 | 2.84 | 7.79 | 75.00 | 2.75 | 132.37 |
| 76 | 1.32 | 7.72 | 35.11 | 2.80 | 79.09 |
| 77 | 2.00 | 7.77 | 50.78 | 1.90 | 121.63 |
| 78 | 0.39 | 7.95 | 20.00 | 2.90 | 11.32 |
| 79 | 0.21 | 7.04 | 17.77 | 2.72 | 10.14 |
| 80 | 1.28 | 5.94 | 47.89 | 2.83 | 137.61 |
| 81 | 0.68 | 6.07 | 26.00 | 2.23 | 50.00 |
| 82 | 0.19 | 6.77 | 30.22 | 2.26 | 14.89 |
| 83 | 0.65 | 5.93 | 16.89 | 2.75 | 36.11 |
| 84 | 0.47 | 6.17 | 22.00 | 1.94 | 23.32 |
| 85 | 3.03 | 5.52 | 37.45 | 4.77 | 523.53 |
| 86 | 1.19 | 6.44 | 30.11 | 3.83 | 271.25 |
| 87 | 0.75 | 7.23 | 57.12 | 1.99 | 35.97 |
| 88 | 0.56 | 7.34 | 62.44 | 4.04 | 22.13 |
| 89 | 0.63 | 7.57 | 66.34 | 3.44 | 19.30 |
| 90 | 1.14 | 7.44 | 21.00 | 2.87 | 43.77 |
| 91 | 0.40 | 8.38 | 7.77 | 0.64 | 53.80 |
| 92 | 0.22 | 7.48 | 2.22 | 0.41 | 28.08 |
| 93 | 1.26 | 7.62 | 24.44 | 3.15 | 67.65 |
| 94 | 1.02 | 5.32 | 19.44 | 2.51 | 45.31 |
| 95 | 0.45 | 6.59 | 21.66 | 2.74 | 37.36 |
| 96 | 1.21 | 5.57 | 16.11 | 2.83 | 62.16 |
| 97 | 0.23 | 7.28 | 12.33 | 1.59 | 22.87 |

**(e)** SIC contribution to $CO_2$ efflux ($f_{SIC}$).

| Number | SIC (%) | $f_{SIC}$ (%) | Select or not |
|---|---|---|---|
| 1 | 0.00 | 0 | √ |
| 2 | 0.00 | 0 | √ |
| 3 | 0.00 | 0 | √ |
| 4 | 0.00 | 0 | √ |
| 5 | 0.00 | 0 | √ |
| 6 | 0.00 | 0 | √ |
| 7 | 0.00 | 0 | √ |
| 8 | 0.00 | 0 | √ |
| 9 | 0.00 | 0 | √ |
| 10 | 0.00 | 0 | √ |
| 11 | 0.00 | 0 | √ |
| 12 | 0.00 | 0 | √ |
| 13 | 0.00 | 0 | √ |
| 14 | 0.00 | 0 | √ |
| 15 | 0.00 | 0 | √ |
| 16 | 0.00 | 0 | √ |
| 17 | 0.00 | 0 | √ |
| 18 | 0.00 | 0 | √ |
| 19 | 0.00 | 0 | √ |
| 20 | 0.38 | 0 | √ |
| 21 | 2.03 | 34 | × |
| 22 | 0.26 | 0 | √ |
| 23 | 1.01 | 5 | √ |
| 24 | 0.31 | 6 | √ |
| 25 | 3.14 | 24 | × |
| 26 | 1.86 | 20 | × |
| 27 | 3.03 | 14 | √ |
| 28 | 0.71 | 26 | × |
| 29 | 1.22 | 21 | × |
| 30 | 0.37 | 7 | √ |
| 31 | 0.47 | 25 | × |
| 32 | 0.22 | 0 | √ |
| 33 | 0.77 | 21 | × |
| 34 | 3.65 | 18 | × |
| 35 | 0.16 | 5 | √ |
| 36 | 0.39 | 0 | √ |
| 37 | 0.27 | 13 | × |
| 38 | 1.26 | 29 | × |

| | | | |
|---|---|---|---|
| 39 | 1.54 | 29 | × |
| 40 | 1.41 | 19 | × |
| 41 | 1.45 | 25 | × |
| 42 | 0.00 | 0 | × |
| 43 | 0.64 | 0 | √ |
| 44 | 1.39 | 9 | √ |
| 45 | 1.26 | 16 | × |
| 46 | 0.70 | 0 | √ |
| 47 | 0.60 | 0 | √ |
| 48 | 0.69 | 0 | √ |
| 49 | 0.51 | 9 | √ |
| 50 | 1.41 | 27 | × |
| 51 | 1.79 | 11 | √ |
| 52 | 0.99 | 9 | √ |
| 53 | 0.94 | 10 | √ |
| 54 | 1.42 | 13 | √ |
| 55 | 0.00 | 0 | √ |
| 56 | 0.00 | 0 | √ |
| 57 | 0.00 | 0 | √ |
| 58 | 1.01 | 12 | √ |
| 59 | 1.28 | 0 | √ |
| 60 | 0.00 | 0 | √ |
| 61 | 0.00 | 0 | √ |
| 62 | 0.86 | 0 | √ |
| 63 | 0.00 | 0 | √ |
| 64 | 0.28 | 0 | √ |
| 65 | 0.73 | 8 | √ |
| 66 | 0.39 | 9 | √ |
| 67 | 0.14 | 0 | √ |
| 68 | 0.00 | 0 | √ |
| 69 | 0.00 | 0 | √ |
| 70 | 0.00 | 0 | √ |
| 71 | 0.00 | 0 | √ |
| 72 | 0.25 | 0 | √ |
| 73 | 6.95 | 27 | × |
| 74 | 1.13 | 0 | √ |
| 75 | 2.28 | 0 | √ |
| 76 | 2.24 | 0 | √ |
| 77 | 2.65 | 0 | √ |
| 78 | 0.00 | 0 | √ |
| 79 | 0.00 | 0 | √ |
| 80 | 0.00 | 0 | √ |

| | | | |
|---|---|---|---|
| 81 | 0.00 | 0 | √ |
| 82 | 0.00 | 0 | √ |
| 83 | 0.00 | 0 | √ |
| 84 | 0.00 | 0 | √ |
| 85 | 0.00 | 0 | √ |
| 86 | 0.00 | 0 | √ |
| 87 | 2.66 | 0 | √ |
| 88 | 4.20 | 1 | √ |
| 89 | 0.10 | 0 | √ |
| 90 | 0.47 | 0 | √ |
| 91 | 0.74 | 0 | √ |
| 92 | 0.00 | 0 | √ |
| 93 | 0.00 | 0 | √ |
| 94 | 0.00 | 0 | √ |
| 95 | 0.00 | 0 | √ |
| 96 | 0.00 | 0 | √ |
| 97 | 0.00 | 0 | √ |

**Extended Data Table 9 | Results of the linear mixed-effects regression analyzing $\Delta^{14}C$ of bulk SOC of 97 sites from 10 regions in relation to climate, vegetation, and soil factors.** Linear mixed-effects regression models include climate variables (aridity [1 – aridity index] and mean annual temperature [MAT]), vegetation variables (net primary productivity [NPP], plant cover, and species richness [Spec. richness]), and soil properties (SOC, pH, clay + silt content [Clay + silt], oxalate-extractable Fe and Al oxides [Fe + Al], and microbial respiration rate [Resp. rate]). Region was included as a random effect. Marginal $R^2$ (fixed effects) = 0.55, and conditional $R^2$ (fixed and random effects) = 0.60.

| Parameter | Estimate | Std Error | *t* value | *P* value |
|---|---|---|---|---|
| Intercept | -0.007 | 0.103 | -0.065 | 0.952 |
| Aridity | -0.211 | 0.103 | -2.062 | **< 0.05** |
| MAT | 0.050 | 0.137 | 0.363 | 0.724 |
| NPP | 0.306 | 0.091 | 3.351 | **< 0.01** |
| Plant cover | 0.024 | 0.096 | 0.253 | 0.801 |
| Spec. richness | 0.050 | 0.084 | 0.589 | 0.558 |
| SOC | 0.278 | 0.110 | 2.521 | **< 0.05** |
| pH | -0.076 | 0.135 | -0.563 | 0.575 |
| Clay + silt | -0.024 | 0.098 | -0.246 | 0.807 |
| Fe + Al | 0.104 | 0.098 | 1.069 | 0.290 |
| Resp. rate | 0.066 | 0.105 | 0.630 | 0.530 |

**Extended Data Table 10 | Results of the linear mixed-effects regression analyzing $\Delta^{14}C$ of respired $CO_2$ of 80 sites from 9 regions in relation to climate, vegetation, and soil factors.** Linear mixed-effects regression models include climate variables (aridity [1 – aridity index] and mean annual temperature [MAT]), vegetation variables (net primary productivity [NPP], plant cover, and species richness [Spec. richness]), and soil properties (SOC, pH, clay + silt content [Clay + silt], oxalate-extractable Fe and Al oxides [Fe + Al], and microbial respiration rate [Resp. rate]). Region was included as a random effect. Marginal $R^2$ (fixed effects) = 0.49, and conditional $R^2$ (fixed and random effects) = 0.63.

| Parameter | Estimate | Std Error | *t* value | *P* value |
|---|---|---|---|---|
| Intercept | 0.065 | 0.150 | 0.433 | 0.680 |
| Aridity | -0.224 | 0.116 | -1.938 | 0.057 |
| MAT | 0.027 | 0.160 | 0.170 | 0.867 |
| NPP | 0.136 | 0.090 | 1.514 | 0.135 |
| Plant cover | -0.071 | 0.104 | -0.678 | 0.500 |
| Spec. richness | 0.134 | 0.107 | 1.245 | 0.217 |
| SOC | 0.475 | 0.125 | 3.785 | **< 0.001** |
| pH | 0.109 | 0.142 | 0.771 | 0.443 |
| Clay + silt | -0.043 | 0.129 | -0.329 | 0.743 |
| Fe + Al | 0.170 | 0.116 | 1.473 | 0.146 |
| Resp. rate | -0.057 | 0.120 | -0.477 | 0.635 |

**Extended Data Table 11 | Results of the linear mixed-effects regression analyzing the differences in Δ$^{14}$C of bulk SOC and respired $CO_2$ of 80 sites from 9 regions in relation to climate, vegetation, and soil factors.** Linear mixed-effects regression models include climate variables (aridity [1 – aridity index] and mean annual temperature [MAT]), vegetation variables (net primary productivity [NPP], plant cover, and species richness [Spec. richness]), and soil properties (SOC, pH, clay + silt content [Clay + silt], oxalate-extractable Fe and Al oxides [Fe + Al], and microbial respiration rate [Resp. rate]). Region was included as a random effect. Marginal $R^2$ (fixed effects) = 0.34, and conditional $R^2$ (fixed and random effects) = 0.40.

| Parameter | Estimate | Std Error | *t* value | *P* value |
|---|---|---|---|---|
| Intercept | -0.025 | 0.138 | -0.178 | 0.868 |
| Aridity | 0.289 | 0.150 | 1.929 | 0.059 |
| MAT | -0.011 | 0.176 | -0.062 | 0.951 |
| NPP | -0.358 | 0.126 | -2.839 | **< 0.01** |
| Plant cover | 0.056 | 0.130 | 0.434 | 0.666 |
| Spec. richness | 0.130 | 0.129 | 1.011 | 0.316 |
| SOC | -0.055 | 0.142 | -0.389 | 0.699 |
| pH | 0.112 | 0.174 | 0.642 | 0.524 |
| Clay + silt | -0.303 | 0.174 | -1.743 | 0.087 |
| Fe + Al | 0.036 | 0.129 | 0.279 | 0.782 |
| Resp. rate | -0.088 | 0.146 | -0.600 | 0.551 |

**Extended Data Table 12 | Variance inflation factor (VIF) values for predictor variables included in the linear mixed-effects regression model.** Linear mixed-effects regression models include climate variables (aridity [1 – aridity index] and mean annual temperature [MAT]), vegetation variables (net primary productivity [NPP], plant cover, and species richness [Spec. richness]), and soil properties (SOC, pH, clay + silt content [Clay + silt], oxalate-extractable Fe and Al oxides [Fe + Al], and microbial respiration rate [Resp. rate]). $\Delta^{14}C$ of bulk SOC, $n$ = 97. $\Delta^{14}C$ of respired $CO_2$, $n$ = 80. The difference in $\Delta^{14}C$ of bulk SOC and of respired $CO_2$, $n$ = 80.

| | **bulk $\Delta^{14}C$** | **respired $\Delta^{14}C$** | **difference in $\Delta^{14}C$** |
|---|---|---|---|
| MAT | 2.02 | 2.03 | 2.03 |
| Aridity | 2.04 | 2.12 | 2.12 |
| NPP | 1.64 | 1.40 | 1.40 |
| Plant cover | 1.64 | 1.50 | 1.50 |
| Spec. richness | 1.29 | 1.86 | 1.86 |
| SOC | 2.18 | 2.20 | 2.20 |
| pH | 2.68 | 2.44 | 2.44 |
| Clay + silt | 1.50 | 2.15 | 2.15 |
| Fe + Al | 1.60 | 1.76 | 1.76 |
| Resp. rate | 1.92 | 2.08 | 2.08 |

**Extended Data Table 13 | Results of the linear mixed-effects regression testing the main and interactive effects of aridity, NPP, and SOC on $\Delta^{14}C$ values**. (**a**) $\Delta^{14}C$ of bulk SOC, $n = 97$. (**b**) $\Delta^{14}C$ of respired $CO_2$, $n = 80$.

(**a**)

| Parameter | Estimate | Std Error | *t* value | *P* value |
|---|---|---|---|---|
| Intercept | 0.120 | 0.106 | 1.133 | 0.291 |
| Aridity | -0.281 | 0.094 | -3.001 | **< 0.01** |
| NPP | 0.359 | 0.091 | 3.953 | **< 0.001** |
| SOC | 0.395 | 0.097 | 4.084 | **< 0.001** |
| Aridity × NPP | 0.154 | 0.078 | 1.975 | 0.052 |
| Aridity × SOC | 0.029 | 0.098 | 0.294 | 0.770 |
| NPP × SOC | -0.042 | 0.111 | -0.380 | 0.705 |

(**b**)

| Parameter | Estimate | Std Error | *t* value | *P* value |
|---|---|---|---|---|
| Intercept | 0.054 | 0.141 | 0.385 | 0.712 |
| Aridity | -0.209 | 0.094 | -2.229 | **< 0.05** |
| NPP | 0.173 | 0.093 | 1.865 | 0.066 |
| SOC | 0.418 | 0.114 | 3.671 | **< 0.001** |
| Aridity × NPP | 0.134 | 0.083 | 1.608 | 0.112 |
| Aridity × SOC | -0.119 | 0.101 | -1.177 | 0.243 |
| NPP × SOC | 0.004 | 0.109 | 0.035 | 0.972 |

**Extended Data Table 14 | Comparison of Akaike Information Criterion (AIC) values between simple linear models and piecewise regression models for $\Delta^{14}C$-related variables across selected environmental predictors.** AIC values were calculated to assess the relative fit of simple linear models versus piecewise regression models for relationships between $\Delta^{14}C$ values ($\Delta^{14}C_{SOC}$, $\Delta^{14}C_{CO2}$, and $\Delta^{14}C_{CO2} - \Delta^{14}C_{SOC}$) and key environmental variables (aridity, NPP, and SOC content). $\Delta^{14}C_{SOC}$, the $\Delta^{14}C$ of bulk SOC; $\Delta^{14}C_{CO2}$, the $\Delta^{14}C$ of respired $CO_2$; $\Delta^{14}C_{CO2} - \Delta^{14}C_{SOC}$, the difference in $\Delta^{14}C$ of bulk SOC and respired $CO_2$. Lower AIC values indicate better model performance. Piecewise models exhibited lower AIC values, supporting the presence of significant thresholds in these relationships. Although the only exception was $\Delta^{14}C$ of respired $CO_2$ ~ NPP, the small difference ($\Delta$AIC < 1) still suggests comparable model performance and does not contradict the overall pattern.

| | **AIC (linear)** | **AIC (threshold)** |
|---|---|---|
| $\Delta^{14}C_{SOC}$ ~ aridity | 1129.25 | 1103.21 |
| $\Delta^{14}C_{CO2}$ ~ aridity | 812.11 | 809.73 |
| $\Delta^{14}C_{CO2} - \Delta^{14}C_{SOC}$ ~ aridity | 889.67 | 865.72 |
| $\Delta^{14}C_{SOC}$ ~ NPP | 1135.41 | 1125.93 |
| $\Delta^{14}C_{CO2}$ ~ NPP | 821.29 | 822.20 |
| $\Delta^{14}C_{CO2} - \Delta^{14}C_{SOC}$ ~ NPP | 889.22 | 885.86 |
| $\Delta^{14}C_{SOC}$ ~ SOC | 1130.63 | 1123.55 |
| $\Delta^{14}C_{CO2}$ ~ SOC | 772.97 | 767.84 |
| $\Delta^{14}C_{CO2} - \Delta^{14}C_{SOC}$ ~ SOC | 896.32 | 893.48 |

## References


1 Trumbore, S. Radiocarbon and soil carbon dynamics. *Annu. Rev. Earth Planet. Sci.* **37**, 47-66 (2009).

2 Shi, Z. *et al.* The age distribution of global soil carbon inferred from radiocarbon measurements. *Nat. Geosci.* **13**, 555–559 (2020).

3 He, Y. J. *et al.* Radiocarbon constraints imply reduced carbon uptake by soils during the 21st century. *Science* **353**, 1419-1424 (2016).

4 Fekete, I. *et al.* How will a drier climate change carbon sequestration in soils of the deciduous forests of Central Europe? *Biogeochemistry* **152**, 13-32 (2021).

5 Stoner, S. W. *et al.* Soil organic matter turnover rates increase to match increased inputs in grazed grasslands. *Biogeochemistry* **156**, 145-160 (2021).

6 Zomer, R. J., Xu, J. C. & Trabucco, A. Version 3 of the global aridity index and potential evapotranspiration database. *Sci. Data* **9**, 409 (2022).

7 Brady, N. C. & Weil, R. R. *The nature and properties of soils*. (Prentice Hall Upper Saddle River, NJ, 2008).

8 Slessarev, E. W. *et al.* Water balance creates a threshold in soil pH at the global scale. *Nature* **540**, 567-569 (2016).

9 Kayler, Z. E., Kaiser, M., Gessler, A., Ellerbrock, R. H. & Sommer, M. Application of $\delta^{13}$C and $\delta^{15}$N isotopic signatures of organic matter fractions sequentially separated from adjacent arable and forest soils to identify carbon stabilization mechanisms. *Biogeosciences* **8**, 2895-2906 (2011).

10 Rowley, M. C., Grand, S. & Verrecchia, É. Calcium-mediated stabilisation of soil organic carbon. *Biogeochemistry* **137**, 27-49 (2018).

11 Wang, C. & Kuzyakov, Y. Soil organic matter priming: The pH effects. *Glob. Change Biol.* **30**, e17349 (2024).

12 Rakhsh, F., Golchin, A., Al Agha, A. B. & Nelson, P. N. Mineralization of organic carbon and formation of microbial biomass in soil: Effects of clay content and composition and the mechanisms involved. *Soil Biol. Biochem.* **151**, 108036 (2020).

13 Butcher, K. R., Nasto, M. K., Norton, J. M. & Stark, J. M. Physical mechanisms for soil moisture effects on microbial carbon-use efficiency in a sandy loam soil in the western United States. *Soil Biol. Biochem.* **150**, 107969 (2020).

14 Díaz-Martínez, P. *et al.* Vulnerability of mineral-associated soil organic carbon to climate across global drylands. *Nat. Clim. Chang.* **14**, 976-982 (2024).

15 Maestre, F. T. *et al.* Biogeography of global drylands. *New Phytol.* **231**, 540-558 (2021).

16 Middleton, N. & Thomas, D. *World atlas of desertification*. (Arnold for UNEP, London, 1997).

17 Lavallee, J. M., Soong, J. L. & Cotrufo, M. F. Conceptualizing soil organic matter into particulate and mineral-associated forms to address global change in the 21st century. *Glob. Change Biol.* **26**, 261-273 (2020).

18 Zhang, L. *et al.* Mapping global distributions, environmental controls, and

uncertainties of apparent topsoil and subsoil organic carbon turnover times. *Earth Syst. Sci. Data* **17**, 2605-2623 (2025).
